# Feasibility and signals of efficacy of the Type 1 Diabetes Education and Support (T1DES) intervention to improve diabetes distress and glycemic levels among Black young adults with type 1 diabetes compared to standard diabetes education: study protocol for a randomized pilot trial

**DOI:** 10.1186/s40814-026-01776-z

**Published:** 2026-04-01

**Authors:** Teaniese L. Davis, Courtney McCracken, Lawrence Fisher, Ilana Graetz, Joshua Barzilay, Priyathama Vellanki, J. Sonya Haw, Laura Gonzalez Paz, Rachel Wolf, Mackenzie Crawford, Marsha Wright, Fiona Bock, Swathi Sekar

**Affiliations:** 1 Kaiser Permanente Georgia, Center for Research and Evaluation, Atlanta, GA USA; 2 University of California San Francisco, San Francisco, CA USA; 3 Emory University Rollins School of Public Health, Atlanta, GA USA; 4 The Southeast Permanente Medical Group, Atlanta, GA USA; 5 Emory University School of Medicine, Atlanta, GA USA

**Keywords:** Type 1 diabetes, Diabetes distress, Black, Young adults

## Abstract

**Background:**

Diabetes distress, the negative emotional impact of living with diabetes, is associated with suboptimal outcomes, including higher HbA_1c_, poorer self-management, and reduced quality of life, and contributes to disparities between Black and White patients with type 1 diabetes (T1D). Young adults may be particularly vulnerable to diabetes distress. Young Black adults therefore may benefit from tailored, representative, and inclusive interventions. Yet, few interventions have been designed to center the experiences of diabetes distress among Black young adults with T1D.

**Methods:**

OnTrack, an evidence-based diabetes distress group intervention, was adapted with input from an advisory board comprised of Black young adults to be culturally appropriate for Black young adults (ages 18–30 years) with T1D. The adapted intervention, called Type 1 Diabetes Education and Support (T1DES), will be tested in this pilot randomized trial to assess the feasibility of T1DES in two distinct healthcare systems: an integrated care system and a safety net care system. Participants will be stratified by site and randomized into the intervention condition (T1DES, *n* = 40, 20 per site) or an attention control condition providing a traditional diabetes education program not focused on distress (StreamLine, *n* = 40, 20 per site). Both conditions will include five workshop sessions over 3 months. Feasibility outcomes will include an assessment of intervention acceptability, demand, practicality, fidelity, and economic feasibility. Signals of efficacy will be assessed by changes in diabetes distress (self-reported), diabetes management skills (self-reported), and glycemic control (HbA_1c_) between study arms, which will be collected at baseline and 3 months post intervention and 6 months post intervention.

**Discussion:**

Black young adults with T1D face unique needs and challenges that need to be considered when providing diabetes support. Interventions that tailor content to represent the experiences of Black young adults have the potential to reduce diabetes distress and HbA_1c_ and improve diabetes management. If successful, the T1DES intervention could be disseminated to support the long-term goal of improving diabetes outcomes and reducing healthcare disparities in this population.

**Trial registration:**

ClinicalTrials.gov NCT05735340. Registered on 2023-2-9.

ClinicalTrials.gov NCT06494722. Registered on 2024-7-9.

**Supplementary Information:**

The online version contains supplementary material available at 10.1186/s40814-026-01776-z.

## Introduction {6a}

Type 1 diabetes (T1D) impacts 5–10% of the nearly 30 million people with diabetes in the USA. Patients with T1D have higher healthcare utilization and almost four times more healthcare spending per capita compared to their non-diabetic counterparts [1, 2]. Achieving and sustaining target HbA_1c_ levels is critical for preventing or delaying serious complications, including cardiovascular disease, neuropathy, and retinopathy [3, 4]. Despite T1D being more prevalent among White populations, Black populations are more likely to have poorer glycemic control and higher rates of preventable complications [5] highlighting a pervasive racial disparity. Moreover, glycemic control in T1D is at its lowest among adolescents 18–25 years, with only 14% reaching their target HbA_1c_, compared to other age groups [5]. Thus, young Black adults with T1D represent a population highly vulnerable to poor diabetes outcomes.

Diabetes distress, the negative emotional effect of living with diabetes, is related to the additional diabetes-specific stressors and emotional burdens experienced when living with diabetes [6]. It is a distinct condition compared to depression and is associated with suboptimal HbA_1c_ levels as well as other challenges, including poorer self-management and reduced quality of life [7–9]. Severe diabetes distress is especially common among individuals with T1D [10] and is one of the largest contributors to racial disparities between Black and White young adults ages 18–25 years with T1D. Diabetes management in social situations can be burdensome and can trigger diabetes distress [11, 12]. Young adults with T1D require different types of support to enhance their ability to manage diabetes in a social context [13] and problem-solving skills to adapt their diabetes management routine when daily routines shift [14]. Formative work indicates Black adolescents and young adults with diabetes face unique challenges monitoring glucose, finding jobs to accommodate health needs, feeling isolated, transitioning to adult health care practices, and having more independence managing their diabetes care, paying for care, and educating others about T1D, all of which may contribute to diabetes distress, and that patients preferred supportive diabetes care tailored to meet their specific developmental stage and barriers [15].

A limited number of behavioral interventions have effectively improved diabetes stress and HbA1c for individuals with T1D [16–21]. These interventions were tested primarily among White patients [22–25] or did not report race. More representative, culturally competent [26–30], evidence-based interventions are needed to improve outcomes to help reduce race-based outcome disparities among people living with T1D [31–33]. OnTrack is one intervention that has demonstrated improved diabetes distress and HbA_1c_ among adults with T1D and elevated HbA_1c_ through highly structured, group-based sessions and individual support [23]. However, like other interventions, its relevance to Black young adults has not been tested, and no prior T1D interventions targeting diabetes distress have been tailored and tested for young Black adults with T1D [34–36].

### Objectives {6b, 7, 8, 9}

This protocol aligns with the SPIRIT Checklist criteria as outlined by the SPIRIT Group [37]. This paper presents the protocol for testing the adaptation and implementation of the OnTrack behavioral intervention program with a focus on managing diabetes distress for young Black adults with T1D in a randomized, two-arm pilot trial. Participants will be allocated 1:1 to either the adapted intervention, called Type 1 Diabetes Education and Support (T1DES), or to StreamLine, a traditional diabetes education program that will serve as an attention control. The primary aim of this study is to evaluate the feasibility (intervention acceptability, demand, practicality, and implementation fidelity, Table 2) [38] of the culturally tailored intervention (T1DES) across two healthcare settings: an integrated health system serving patients within metropolitan Georgia and a public hospital system providing primarily indigent care to underserved populations in Atlanta. The secondary aim of the study is to evaluate the signals of efficacy of T1DES on diabetes distress and glycemic control among these young Black adults.

## Methods

The T1DES study began in April 2022 with funding from the National Institute of Diabetes and Digestive and Kidney Diseases (1R01DK128236-01A1) with recruitment starting in March 2023 {4}. Additional funding to add a public hospital system as a new recruitment site was received in May 2023 from the Helmsley Charitable Trust (G-2310–06383) {4} with recruitment starting in March 2024.

Funding for the study was provided by grants from the National Institutes of Health, National Institute of Diabetes and Digestive and Kidney Diseases (NIDDK), and The Leona M. and Harry B. Helmsley Charitable Trust. All study protocols will be reviewed and approved by the Institutional Review Board (IRB) and will follow all compliance rules for protecting research participants’ confidentiality. The study will also obtain a Certificate of Confidentiality to provide special protection for participants involved in clinical research.

### Study design {8}

**Fig. 1:**
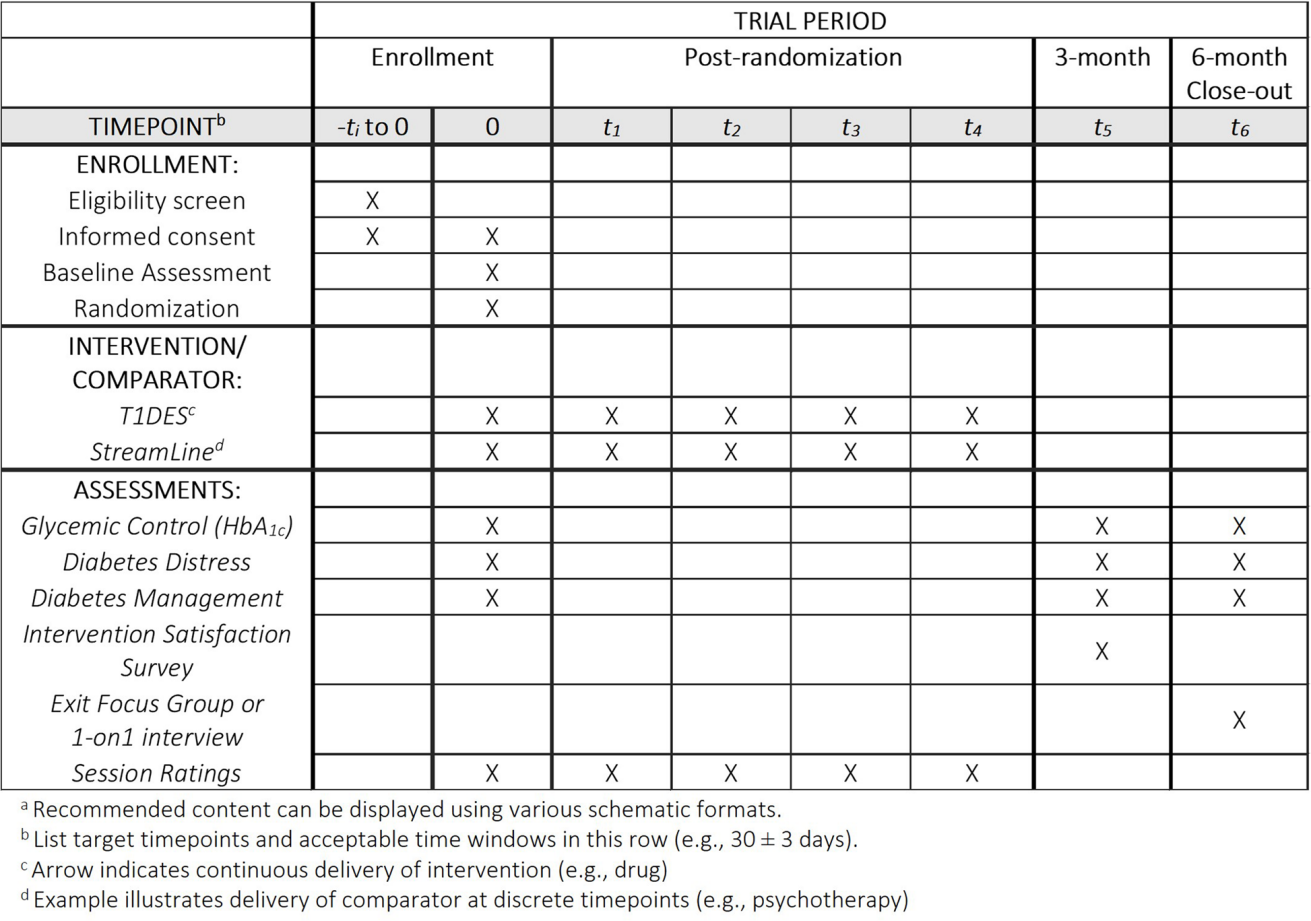
SPIRIT 2025 participant timeline: schedule of enrollment, interventions, and assessments^a^

This study is a two-arm, pilot, randomized clinical trial (RCT) using convergent parallel mixed methods to evaluate the feasibility (Aim 1) and signals of efficacy of T1DES on diabetes outcomes (Aim 2). The study duration is 6 months; 3 months of intervention with an additional 3 months of follow-up. At baseline (T_1_) participants will complete a quantitative survey, complete a point of care HbA_1C_ test, and session 1 (group-based session). Sessions 2–5 will occur virtually over the following 3 months at 2–3 week intervals. At 3 months post-baseline (T_2_), after the last intervention and standard of care group sessions, participants will complete the session satisfaction survey, quantitative survey, and point of care HbA_1c_. At 6 months post-baseline (T_3_), participants will complete the quantitative survey and point of care HbA_1c_ followed by exit focus groups (Fig. 1—T1DES SPIRIT consort and timeline).

### Participants {10}

Individuals will be eligible to participate in the study if they are patients of Grady Health System (Grady) or Kaiser Permanente Georgia (KPGA) at the time of study enrollment and:

- Aged 18–30 years
- Self-reported race of Black or African American
- Confirmed diagnosis of T1D
- HbA_1c_ > 7.5 in the past 12 months
- Able to read in English and provide informed consent

Individuals will be excluded if they have a developmental delay or other cognitive impairment that would render them unable to provide informed consent, visual impairment, or have severe hearing or other physical disabilities that would be a barrier to participating in-group or web sessions. If eligible, study staff will review the consent form {32} (Supplement 1) with the eligible participant who will then provide a digital signature that is stored in the REDCap database {26a}. Enrolled participants will receive compensation for their time, detailed in Table 1.

**Table 1:**
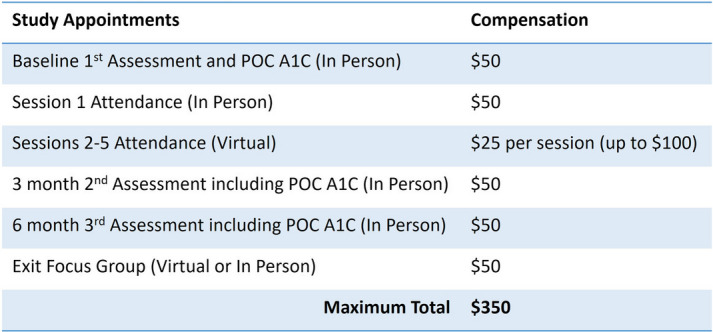
Participant compensation

### Recruitment and retention methods {11c, 15, 18b}

Participants will be recruited using multiple methods (Table 2—T1DES recruitment strategies). (1) A pre-identified contact list of potentially eligible members will be generated based on the data abstracted from the electronic medical record (EMR) at each health system. This information will be entered into REDCap {27}. This list will be used to contact individuals via email, phone, and short message service (SMS) text messages. Email and text communications will be automatically sent through REDCap using Twilio, which allows programmable text messages within REDCap; (2) printed materials with a QR code will be available at each site and broadcast on the internal television screens in-clinic. Flyers and television messaging at these locations will direct interested individuals to contact the research team via the main study line or an online interest form; (3) endocrinology providers at each site will be asked to inform members about the T1DES study during appointment reminders, as well as virtual and in-person clinical visits; (4) in-person recruitment during specialty clinic hours serving patients with T1D.

**Table 2:**
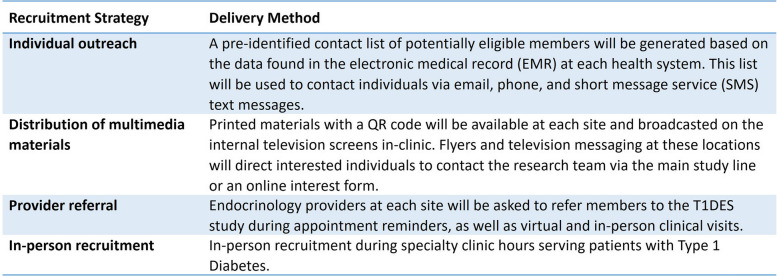
TIDES recruitment strategies

Once contacted, staff will provide potentially eligible participants with a study overview, confirm the individual’s interest, and complete a screening questionnaire (Table 3). Due to the inherent nature of missing or incorrect data in the EMR, potentially eligible individuals will still be screened via phone to confirm eligibility.

**Table 3:**
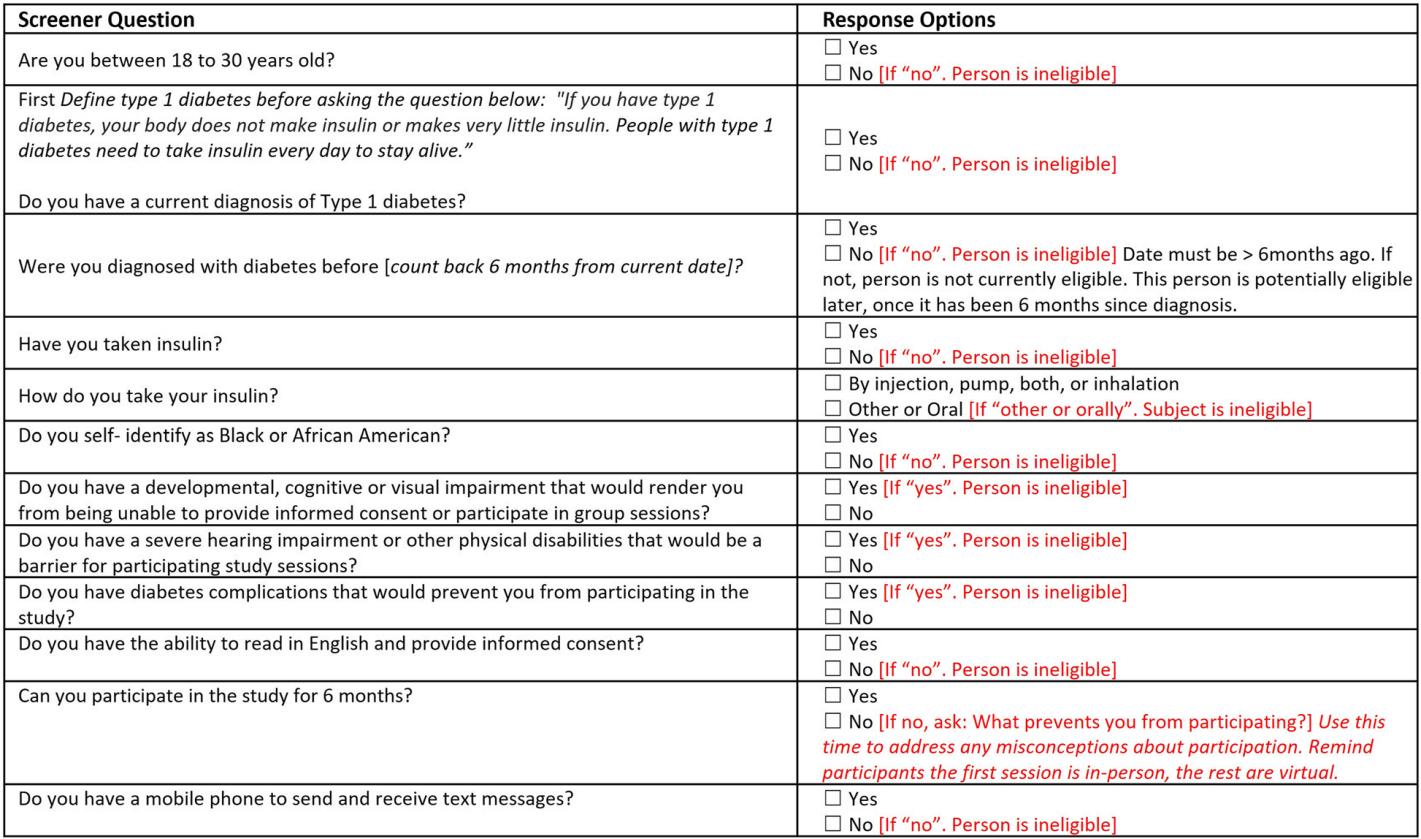
Screening questions to determine study eligibility across sites

If eligible, participants will be invited to attend the next baseline session. Participants will be recruited in groups since this is a group-based intervention. If someone meets all eligibility criteria but does not have an HbA_1c_ in their EMR in the past 12 months, they will be invited to arrive early to the upcoming baseline session and complete a point-of-care HbA_1c_. There will be a separate consent form for the HbA_1c_ pre-screening to confirm study eligibility. They will receive $10 for completing the HbA_1c_. If the POC HbA_1c_ is > 7.5, the person will be given the consent form for the T1DES Study. If POC HbA_1c_ ≤ 7.5, they will not be eligible for the trial but will be provided with resources on various diabetes topics including, but not limited to, time in range, carb counting, work and school accommodations, and socializing with T1D. Cohorts will be recruited approximately bimonthly. Eligible participants will be invited to attend one of the predefined start dates (baseline) for each cohort.

**Fig. 2:**
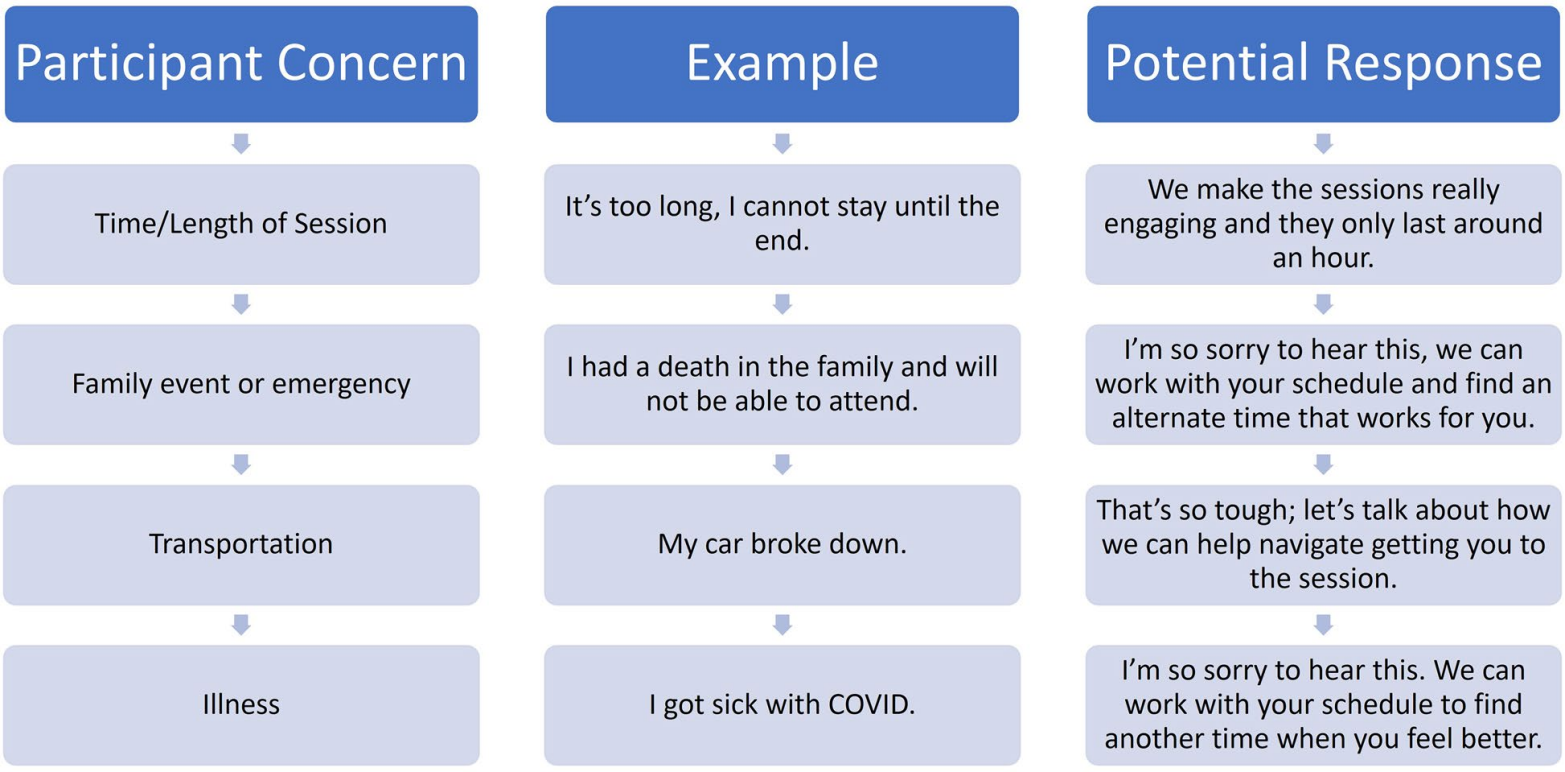
Application of AASAP (Anticipate, Acknowledge, Standardize, Accept, and Plan) method for recruiting and retaining participants

Study retention methods include phone calls, postcards sent via U.S. mail, text messages, and emails. The team will be trained to use the Anticipate, Acknowledge, Standardize, Accept, and Plan (AASAP) method when making phone calls. AASAP [39] is an effective 5-step strategy for recruiting and retaining hard-to-reach populations (Fig. 2—application of the AASAP). AASAP uses motivational interviewing during recruitment and retention calls with participants. Staff are trained to reflect on what the participants’ concerns are regarding their attendance at upcoming appointments and guide participants to consider alternatives. For example, if a participant cannot attend due to childcare or work, staff acknowledge the issue and ask if there can be alternate accommodations made to allow the member to attend.

### Assignment to condition {16a, 16b, 16c, 17a, 17b}

We will randomize N = 80 participants to either the T1DES intervention (*n* = 40) or StreamLine standard of care (*n* = 40) condition. Randomization will be stratified by site (KPGA and Grady), and intervention conditions will be balanced between strata using random permuted blocks of size 2, 4, and 8 to construct the randomization assignments. Upon completing consenting procedures and the baseline (T1) assessment, participants will be randomized. Randomization assignments will be performed by the study statistician and Co-I (McCracken). Pre-randomized sealed envelopes will be given to study participants by study staff after completing all baseline assessments. The statistician remains blinded to individuals’ assignments. There is no foreseeable reason for unblinding.

### Intervention (T1DES) {11a}

The T1DES intervention was adapted from an evidence-based intervention for individuals with T1D (OnTrack) guided by the ADAPT-ITT model (Table 4). OnTrack was selected because it is an evidence-based, behavioral intervention that demonstrated improved outcomes in diabetes distress and glycemic control among adults (mean age 45.1 years, SD = 15.0) with T1D and elevated HbA_1c_ (> 7.5). It included highly structured, group-based sessions (facilitated by a counselor) and individual support [23]. OnTrack used scenarios and exercises to assist with emotion regulation in individuals with T1D. OnTrack resulted in greater reductions in diabetes distress among participants who had lower cognitive function or emotion regulation, higher baseline distress, and greater diabetes knowledge.

**Table 4:**
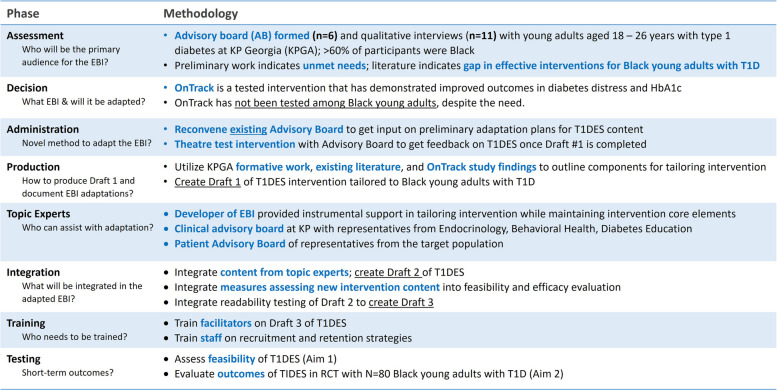
ADAPT-ITT model for tailoring an evidence-based intervention (OnTrack) for Black young adults T1D

Preliminary feedback from previous advisory board members (ADAPT-ITT Administration Phase) and qualitative interviews with young adults with T1D indicated this intervention would require some modifications to make the content relevant and appropriate to Black young adults. Key highlights of the adaptation included replacing content with new topics including reproductive health, intersectionality, and use of diabetes technology. Images and storyline protagonists used throughout the intervention will be changed to resonate with the new audience. Additional details about the intervention adaptation will be published separately to describe the full intervention adaptation process.

While the tailored intervention (T1DES) will include new elements such as scenarios representative of Black young adults’ lives and adding SMS-based information and support, the core elements from OnTrack will remain. Participants will participate in a 3-month intervention including 5 one-hour group sessions, individual check-ins before each group session, and SMS-delivered T1DES intervention content. Before each group session, facilitators will contact participants individually to virtually check in with participants about the upcoming session and remind participants to complete the session homework.

**Fig. 3:**
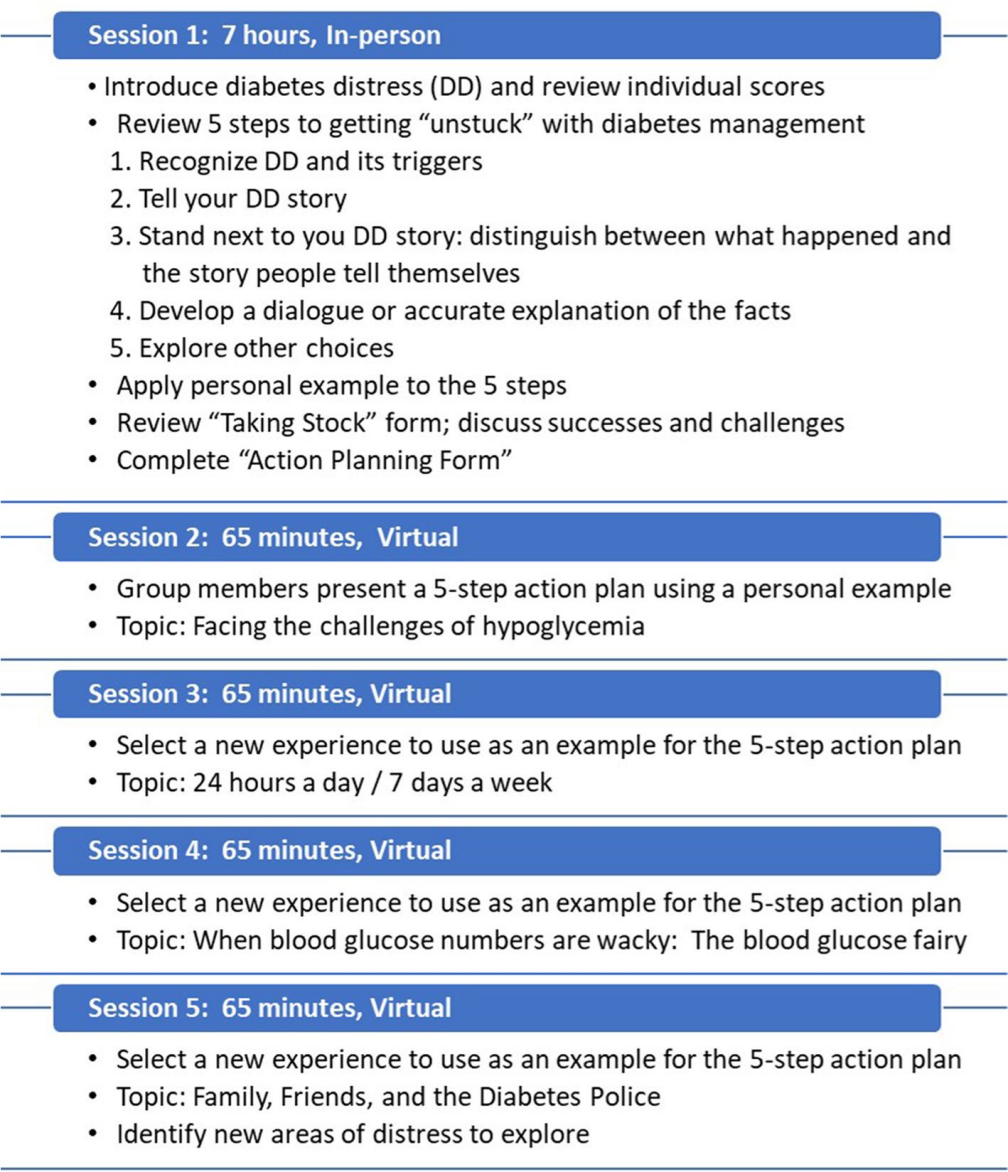
Description of T1DES group sessions

Intervention techniques stem from motivational interviewing [40], and empowerment-based communication [41–43]. It incorporates scenarios and activities to help participants cope with the emotional components of having T1D and is based on emotion regulation. T1DES helps participants develop personalized emotion management techniques, which enable them to make behavioral changes important for diabetes self-management [35]. T1DES sessions will be led by a licensed clinical therapist. Multiple therapists will be hired to rotate group facilitation. Figure 3 provides additional details about T1DES intervention content. During session 1, participants in T1DES will develop a personalized action plan and outline the positive and negative feelings associated with each behavior change. At the follow-up sessions (2–5), facilitators follow up with participants on their personalized action plan, as well as discuss different topics at each session.

Session 2 focuses on family, friends, intimate relationships, and starting advocacy with people close to you before transitioning to the school, work, and medical setting.

Session 3 focuses on T1D Advocacy in school, work, and health care. This will be a merged discussion to focus on hypoglycemia and blood glucose numbers. Communicating with doctors and navigating healthcare to facilitate enhanced responses and communication with providers.

Session 4 focuses on diabetes management being 24 h a day, 7 days a week and discusses facing diabetes burnout and taking a diabetes vacation. Participants discuss how diabetes burnout presents for each person; how to handle when it feels like technology or devices are not working or are failing you, which will transition you into handling distress; and being able to take a “naked” shower and the relief or sense of freedom in the naked shower.

Session 5 focuses on handling mental health challenges of your diagnosis, including anxiety and depression; discussing how this impacts metrics.

### Standard of care (StreamLine) {11a}

StreamLine, the standard of care condition, will be 5, 30-min, diabetes education-only sessions facilitated by trained Diabetes Educators. StreamLine will follow the standard of care model which offers diabetes education sessions for members with diabetes. In addition, the endocrinology department offers links to resources both internal and external to KPGA and Grady. These include connecting members with various departments and resources on the intranet to help navigate healthcare at KPGA and Grady, pamphlets produced by external sources such as the American Diabetes Association and The Diabetes Link, and links to online sources of information and support. The resources provided vary by provider. Therefore, for this study, we will standardize the resources traditionally provided at KPGA and Grady for the diabetes education-only control condition. Like the T1DES condition, we will hire multiple Diabetes Educators to rotate group facilitation.

Session 1 will be in-person and sessions 2–5 will be virtually delivered individual sessions. StreamLine focuses on defining HbA_1c_, health and nutrition recommendations, diabetes management tools, navigating hypo- and hyperglycemia, and diabetes complications. The standard of care arm was not adapted; however, our study team made important updates to the sessions including updating the following items: (1) resources to be specific to the Georgia region, (2) information on continuous glucose monitoring (CGM), and (3) diabetes management options.

### Outcomes measures {12,14, 18a}

The primary outcome is feasibility, and the secondary outcome is preliminary intervention signals of efficacy [44]. Table 5 details the measures for evaluating feasibility [45] (Aim 1) and signals of efficacy (Aim 2) of T1DES; the constructs outlined align with the RE-AIM framework [38, 46] RE-AIM is a model widely used to measure disease management intervention feasibility and signals of efficacy [45].

**Table 5:**
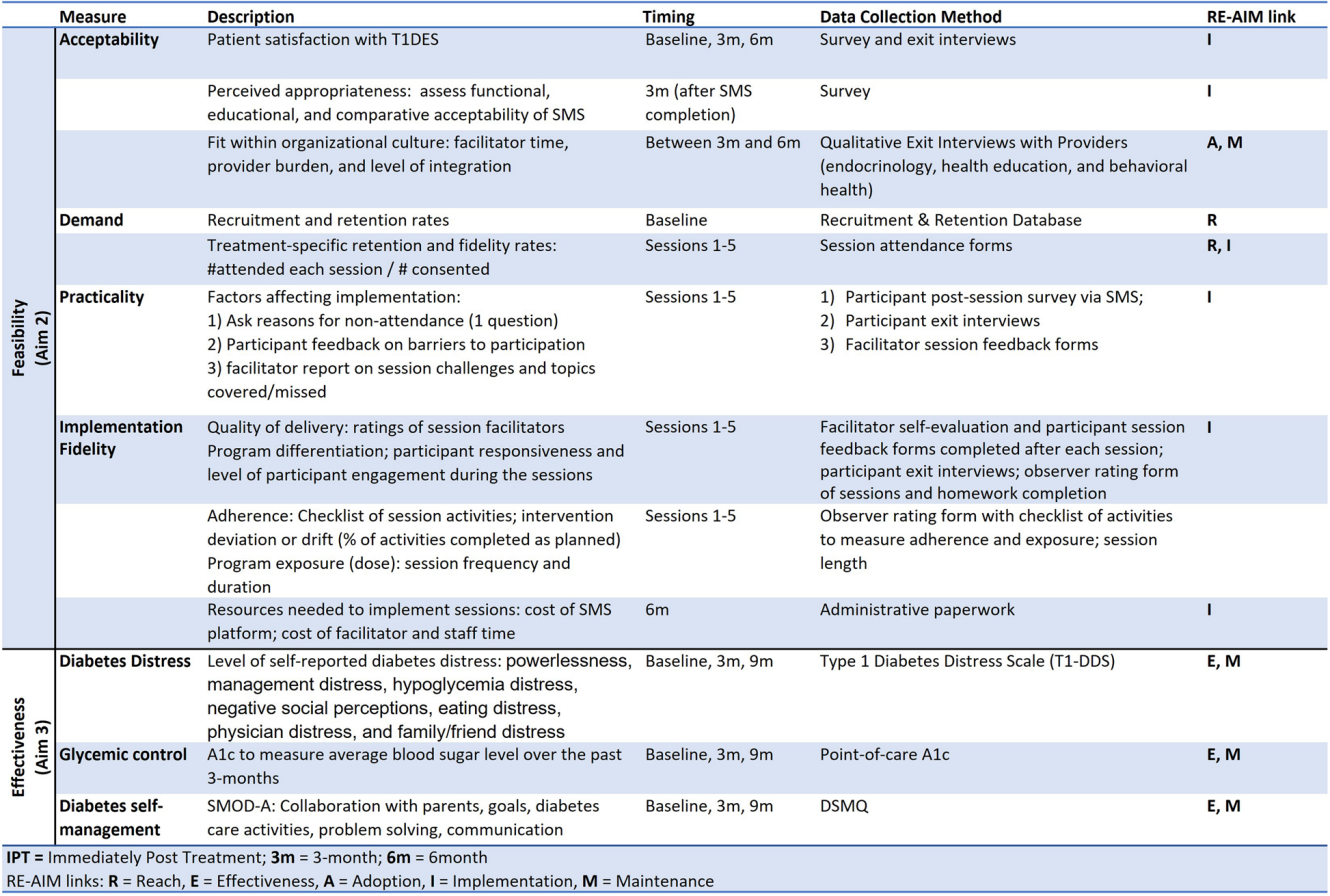
Feasibility and effectiveness study measures for evaluating T1DES Intervention linked to RE-AIM framework

Feasibility (Aim 1): Assess the feasibility of T1DES by measuring intervention acceptability (satisfaction, perceived appropriateness), demand (uptake, retention > 80%), practicality (factors affecting implementation), economic feasibility (costs of implementation and lost productivity), and implementation (degree of execution; intervention fidelity) through quantitative surveys, key informant focus groups, and one-on-one interviews with participants and the health care delivery team [47, 48]. Measures are outlined separately in Appendices D–J. 

Signals of Efficacy (Aim 2): Evaluate signals of efficacy of T1DES on diabetes by comparing the changes in HbA_1c_, diabetes distress, and self-management behaviors from baseline to 6 months post-baseline among participants randomized to the T1DES intervention compared to the StreamLine standard of care condition based on intent to treat. At all timepoints (T_1_–T_3_), we will measure HbA_*1c,*_ diabetes distress (*Type 1 Diabetes Distress (T1-DDS))* [49, 50]*,* and *Diabetes Self-Management (DSMQ) * [51].

*Type 1 Diabetes Distress (T1-DDS)* [50, 52] is a 28-item scale with seven (7) subscales: powerlessness, management distress, hypoglycemia distress, negative social perceptions, eating distress, physician distress, and family/friend distress. Response options range from 1 (not a problem) to 6 (a very serious problem). Higher scores indicate higher levels of diabetes distress.

*Diabetes Self-Management Questionnaire (DSMQ)* [51] is a 16-item instrument measuring self-care behaviors in five (5) areas in the past eight (8) weeks: dietary control, medication adherence, blood glucose monitoring, physical activity, and physician contact/appointment adherence. Response options range from *does not apply to me* (0) to *applies to me very much* (3). Higher scores indicate more desirable self-care behaviors.

*Biomarker data—HbA*_*1c*_: The HbA_1c_ measures average blood sugar level over the past 3 months; we will measure HbA_1c_ at all timepoints (T_1_-T_3_). A point-of-care (POC) HbA_1c_ blood test will occur at each of these timepoints and will be administered by a trained staff member.

### Sample size {12, 14}

This study will aim to recruit and randomize 80 participants with T1D (40 per study arm and 40 per site), a sample size allowing us to evaluate feasibility [53] and signals of efficacy. Because this is a pilot randomized trial, the sample size is based on feasibility objectives (retention and intervention participation) rather than hypothesis testing for effectiveness outcomes. Consistent with published guidance for pilot trials, the target sample size is selected to provide useful precision for estimating key feasibility parameters and to inform decision-making and planning for a future definitive trial [54, 55]. Our a priori feasibility targets are (1) retention > 80%, defined as completion of the 3-month follow-up survey and A1c assessment, and (2) participation > 80%, defined as attending ≥ 4 of 5 intervention sessions. With 40 participants per arm, the study will provide arm-specific estimates of these feasibility proportions with reasonable precision for decision-making about progression to a fully powered trial (e.g., if 32/40 meet the criterion, the approximate 95% CI around an 80% proportion is ~0.65 to ~0.90) [56].

Our key secondary goal is to evaluate signals of efficacy of T1DES on diabetic outcomes in Black young adults by comparing changes from baseline and to participants randomized to StreamLine (attention control) to those randomized to T1DES. We recognize that this pilot study is underpowered to detect small to moderate differences in outcomes between our intervention groups; nevertheless, data generated from this small R01 pilot study will be essential for generating effect size estimates to use when planning a large-scale RCT {14}. For signals of efficacy, a sample size of 80 participants provides at least 80% power to detect a 1.5-point difference in the *change* in HbA_1C_ (from baseline to 3 or 6 months) between the T1DES group and StreamLine intervention conditions using a two-sided two-sample *t*-test with alpha = 0.05. This results in an effect size of *d* = 0.75. If we experience 10–20% attrition, we will have 80% power to detect effect sizes between 0.78 and 0.83. We recognize that these large effect sizes may not be observable in our trial; however, these data will provide an estimate of the anticipated change in our outcome measures that can be used to adequately power a future large-scale RCT. Power estimates were calculated using PASS v. 14.0.8 (Kaysville, UT).

### Data management {19, 27}

All data obtained from the EMR and through direct participant report (e.g., surveys, focus groups) will be stored on a secure server at either KPGA or Emory in REDCap. REDCap requires unique logins and is HIPAA compliant. All paper research files with PHI will be kept in locked file cabinets in KPGA and Emory University buildings. Study participants will be assigned a unique study ID, which will be used to reduce the need for names and other identifiable information when possible. Data from both study sites will be combined for a total proposed sample size of *N* = 80 participants.

All electronic research files will be kept on a secure server that uses multiple layers of data protection, and all research personnel will sign a confidentiality agreement and receive NIH and CITI human subjects training. Data will be accessible only to research staff, using confidential usernames and passwords. All paper research files will be kept in locked file cabinets in KPGA and Emory University buildings. Survey data will be collected using REDCap with HIPAA-compliant features, which will help safeguard against data breaches and maintain security of participant data.

### Statistical analyses {20a, 20b, 20c}

Demographics and baseline characteristics will be summarized overall and by intervention condition to assess similarity of intervention groups with respect to baseline characteristics. For the primary outcome of feasibility, metrics including participation rates (recruit and consent 43% of the eligible sample), attrition rates (80% participation at 3- and 6-month follow-up assessments), satisfaction, intervention specific attendance rates (80% attendance at T1DES and StreamLine sessions), fidelity rates, and session ratings will be tabulated using descriptive statistics. Likert-scale values will be summarized using counts and percentages or means and standard deviations, as appropriate. Categorical data will be summarized using counts and percentages. Continuous variables will be summarized using means and standard deviations or medians and percentiles based on the distribution of the data. Histograms and density plots will be used to determine whether means or medians should be reported. These metrics will be calculated overall and by intervention group.

Signals of efficacy outcomes (Aim 2) including HbA_1c_, diabetes self-management skills, and diabetes distress will be summarized by intervention condition at all time points. For all models, we will adjust for site and any group characteristics that differ meaningfully at baseline and may be related to the outcomes. The primary analysis will use an intention-to-treat approach, that is using the intervention assignment based on randomization, regardless of participation.

Generalized linear mixed models will be used to examine changes in the signals of efficacy outcome measures over time. Models will include the fixed effects: time (baseline, 3 months, 6 months), intervention condition (T1DES vs. StreamLine), site (Grady, KPGA), and the intervention × time interaction term. The interaction term will be included in all models and will serve as the primary test of efficacy. Intervention differences will be estimated at 3 months and 6 months, conditional on baseline response levels [57]. Results will be presented as model-estimated differences in means with associated 95% confidence intervals. Using stratified analyses, we will explore differences in feasibility measures and signals of efficacy outcomes by recruitment site. Effect sizes will be calculated overall and by intervention site. We will conduct an exploratory, per-protocol analysis of the signals of efficacy outcomes. Per-protocol criteria are defined as participants that attended at least 80% (i.e., 4 out of 5) intervention sessions in the T1DES condition. All statistical tests will be two-sided, and statistical significance will be assessed using 95% confidence intervals. Analyses will be conducted using SAS v. 9.4 (Cary, NC). We will analyze participant exit focus groups and one-on-one interviews using established qualitative data analysis procedures. In addition to identifying emerging codes during open coding, we will include themes linked to diabetes distress, use of motivational interviewing (MI) during sessions, and group interaction. Then during axial coding, the research team will re-read the transcripts, categorizing data into themes from the codebook [58]. Qualitative data will be coded using NVivo 12.0 Pro [59]. Two coders will be trained to reach a minimum inter-coder agreement of 80%. After both coders have completed three (3) transcripts, they will meet with Dr. Davis (PI) to compare codes and identify possible sources of definitional drift or inconsistencies in applying codes [60].

The sources of data above will all inform study feasibility (see Table 5) to understand participants’ experiences in the intervention. Specifically, data from exit focus groups and interviews, quantitative session ratings, qualitative session feedback (participants, facilitators, study raters), and survey data (diabetes distress and diabetes management) will be used to triangulate participants’ experiences in the intervention, as well as feasibility and signals of efficacy of T1DES.

### Reporting adverse events {5d, 21a, 22, 23}

The study will use a data and safety monitoring plan (DSMP) to govern the monitoring of adverse events and other unanticipated problems. Briefly, the DSMP describes the risks and benefits to participants, procedures for monitoring safety, definitions and classifications of adverse events, and timelines for monitoring and reporting events. With that said, this is a minimal risk study that will not use any investigational drugs or medical therapy in our study population and thus we do not anticipate adverse events related to this study. Given this study poses minimal risk, we elect to use an independent medical monitor to review safety events and unanticipated problems. The data monitoring and safety review will occur in conjunction with the review of the DSMP and will occur after the first participant is enrolled and every 6 months thereafter. The team will review recruitment, enrollment, and retention numbers, overall and by site. They will review protocol deviations and reportable events. In a blinded manner, the study statistician will also present adverse events derived from the health record, including hospitalizations and emergency department visits. The Principal Investigator (T. Davis) and Co-Investigator (J. Barzilay) will evaluate any unanticipated adverse events and determine whether the adverse events affect the risk/benefit ratio of the study and whether modifications to the protocol or consent form are required. A summary of any adverse events will be reported to the IRB, at minimum, when annual re-approval of the protocol is sought. All serious adverse events will be collected and reported to the IRB per the KPGA IRB’s guidelines.

## Discussion

This project addresses a critical public health issue, the impact of T1D on young Black adults in the United States. T1D already affects a significant portion of the population, and this study acknowledges the disparities in glycemic control and healthcare outcomes that disproportionately affect Black patients. The T1DES study recognizes the intersectionality of race and age, highlighting how young Black adults have repeatedly discussed feeling doubly disadvantaged in terms of diabetes outcomes and glycemic control during T1DES advisory board meetings. By identifying the unique challenges faced by this population, this research aims to develop a culturally tailored and responsive intervention, T1DES, to address diabetes distress and improve glycemic control [5].

The impact of this work is two-fold. First, it acknowledges and seeks to rectify the existing racial disparities in diabetes management, which is a critical step towards achieving health equity. By focusing on young Black adults with T1D, the study is attentive to a specific demographic not represented in healthcare interventions. This project's potential impact lies in its capacity to address the unmet needs of young Black adults with T1D and improve their diabetes management outcomes. Second, the development and evaluation of the T1DES intervention represent a significant effort to bridge the gap between existing interventions and the needs of the Black young adult population. If successful, this culturally tailored intervention could serve as a model for future programs targeting under-represented communities with chronic health conditions, potentially reducing healthcare disparities and improving overall health outcomes.

## Supplementary Information

Supplementary material 1. Appendix Consent forms.

Supplementary material 2. SPIRIT Checklist.

## Data Availability

The materials used or analyzed during the current study will be made available by the corresponding author upon reasonable request. Data sets collected during the study will be available to project investigators and study staff as necessary. The final password protected study dataset will be stored on the KPGA server. Password protected data sets may be transferred to Co-Investigators and staff using password protected secure file transfers, aligning with data use agreements.

## References

[1] Harris MI. Racial and ethnic differences in health insurance coverage for adults with diabetes. Diabetes Care. 1999;22(10):1679–82. 10526734 10.2337/diacare.22.10.1679

[2] 2016 Health Care Cost and Utilization Report. 2018. Health Care Cost and Utilization Report. June 19, 2018. https://healthcostinstitute.org/hcci-originals-dropdown/all-hcci-reports/2016-health-care-cost-and-utilization-report. Accessed 22 Sept 2023.

[3] Nathan DM, Group DER. The diabetes control and complications trial/epidemiology of diabetes interventions and complications study at 30 years: overview. Diabetes Care. 2014;37(1):9–16. 24356592 10.2337/dc13-2112 PMC3867999

[4] Osborn CY, de Groot M, Wagner JA. Racial and ethnic disparities in diabetes complications in the northeastern United States: the role of socioeconomic status. J Natl Med Assoc. 2013;105(1):51–8. 23862296 10.1016/s0027-9684(15)30085-7 PMC3852686

[5] Miller KM, Foster NC, Beck RW, Bergenstal RM, DuBose SN, DiMeglio LA, et al. Current state of type 1 diabetes treatment in the US: updated data from the T1D Exchange clinic registry. Diabetes Care. 2015;38(6):971–8. 25998289 10.2337/dc15-0078

[6] Dennick K, Sturt J, Speight J. What is diabetes distress and how can we measure it? A narrative review and conceptual model. J Diabetes Complications. 2017;31(5):898–911. 28274681 10.1016/j.jdiacomp.2016.12.018

[7] Hessler D, Fisher L, Polonsky W, Masharani U, Strycker L, Peters A, et al. Diabetes distress is linked with worsening diabetes management over time in adults with type 1 diabetes. Diabet Med. 2017;34(9):1228–34. 28498610 10.1111/dme.13381 PMC5561505

[8] Stahl‐Pehe A, Glaubitz L, Baechle C, Lange K, Castillo K, Toennies T, et al. Diabetes distress in young adults with early‐onset type 1 diabetes and its prospective relationship with HbA1c and health status. Diabet Med. 2019;36(7):836–46. 30761589 10.1111/dme.13931

[9] Balfe M, Doyle F, Smith D, Sreenan S, Brugha R, Hevey D, et al. What’s distressing about having type 1 diabetes? A qualitative study of young adults’ perspectives. BMC Endocr Disord. 2013;13:1–14. 23885644 10.1186/1472-6823-13-25 PMC3733731

[10] Speight J, Browne JL, Holmes-Truscott E, Hendrieckx C, Pouwer F. Diabetes MILES--Australia (management and impact for long-term empowerment and success): methods and sample characteristics of a national survey of the psychological aspects of living with type 1 or type 2 diabetes in Australian adults. BMC Public Health. 2012;12:120. 10.1186/1471-2458-12-120. 10.1186/1471-2458-12-120 PMC3312855 22325032

[11] Agarwal S, Kanapka LG, Raymond JK, Walker A, Gerard-Gonzalez A, Kruger D, et al. Racial-ethnic inequity in young adults with type 1 diabetes. J Clin Endocrinol Metab. 2020;105(8):e2960–9. 32382736 10.1210/clinem/dgaa236 PMC7457963

[12] Polonsky WH, Anderson BJ, Lohrer PA, Welch G, Jacobson AM, Aponte JE, et al. Assessment of diabetes-related distress. Diabetes Care. 1995;18(6):754–60. 7555499 10.2337/diacare.18.6.754

[13] Wiebe DJ, Berg CA, Mello D, Kelly CS. Self-and social-regulation in type 1 diabetes management during late adolescence and emerging adulthood. Curr Diabetes Rep. 2018;18:1–9. 10.1007/s11892-018-0995-3 29564640

[14] Majumder E, Cogen FR, Monaghan M. Self-management strategies in emerging adults with type 1 diabetes. J Pediatr Health Care. 2017;31(1):29–36. 26861574 10.1016/j.pedhc.2016.01.003 PMC4976043

[15] Davis TL, Barzilay JL, Walker-Williams DR, Robinson BR, Rassouli N. A qualitative examination of experiences managing type 1 diabetes among adolescents and young adults aged 18 to 26 years. presented at: 2019 Annual Meeting of the Health Care Systems Research Network (HCSRN); 2019; Portland, Oregon.

[16] Whitehead L, Seaton P. The effectiveness of self-management mobile phone and tablet apps in long-term condition management: a systematic review. J Med Internet Res. 2016;18(5):e97. 27185295 10.2196/jmir.4883 PMC4886099

[17] Louch G, Dalkin S, Bodansky J, Conner M. An exploratory randomised controlled trial using short messaging service to facilitate insulin administration in young adults with type 1 diabetes. Psychol Health Med. 2013;18(2):166–74. 22646659 10.1080/13548506.2012.689841

[18] Huang JS, Terrones L, Tompane T, Dillon L, Pian M, Gottschalk M, et al. Preparing adolescents with chronic disease for transition to adult care: a technology program. Pediatrics. 2014;133(6):e1639–46. 24843066 10.1542/peds.2013-2830 PMC4035589

[19] Cafazzo JA, Casselman M, Hamming N, Katzman DK, Palmert MR. Design of an mHealth app for the self-management of adolescent type 1 diabetes: a pilot study. J Med Internet Res. 2012;14(3):e2058. 10.2196/jmir.2058 PMC3799540 22564332

[20] Farmer AJ, Gibson OJ, Dudley C, Bryden K, Hayton PM, Tarassenko L, et al. A randomized controlled trial of the effect of real-time telemedicine support on glycemic control in young adults with type 1 diabetes (ISRCTN 46889446). Diabetes Care. 2005;28(11):2697–702. 16249542 10.2337/diacare.28.11.2697

[21] Monaghan M, Helgeson V, Wiebe D. Type 1 diabetes in young adulthood. Curr Diabetes Rev. 2015;11(4):239–50. 25901502 10.2174/1573399811666150421114957 PMC4526384

[22] Sturt J, Dennick K, Due-Christensen M, McCarthy K. The detection and management of diabetes distress in people with type 1 diabetes. Curr Diabetes Rep. 2015;15:1–14. 10.1007/s11892-015-0660-z 26411924

[23] Fisher L, Hessler D, Polonsky WH, Masharani U, Guzman S, Bowyer V, et al. T1-REDEEM: a randomized controlled trial to reduce diabetes distress among adults with type 1 diabetes. Diabetes Care. 2018;41(9):1862–9. 29976567 10.2337/dc18-0391 PMC6105321

[24] Bond GE, Burr RL, Wolf FM, Feldt K. The effects of a web-based intervention on psychosocial well-being among adults aged 60 and older with diabetes. Diabetes Educ. 2010;36(3):446–56. 20375351 10.1177/0145721710366758

[25] Berlin KS, Rabideau EM, Hains AA. Empirically derived patterns of perceived stress among youth with type 1 diabetes and relationships to metabolic control. J Pediatr Psychol. 2012;37(9):990–8. 22753443 10.1093/jpepsy/jss080

[26] Joo JY, Liu MF. Culturally tailored interventions for ethnic minorities: a scoping review. Nurs Open. 2021;8(5):2078–90. 34388862 10.1002/nop2.733 PMC8363345

[27] Sukkarieh-Haraty O, Egede LE, Khazen G, Abi Kharma J, Farran N, Bassil M. Results from the first culturally tailored, multidisciplinary diabetes education in Lebanese adults with type 2 diabetes: effects on self-care and metabolic outcomes. BMC Res Notes. 2022;15(39):7. 10.1186/s13104-022-05937-0 PMC8832854 35144687

[28] Utz SW, Williams IC, Jones R, Hinton I, Alexander G, Yan G, et al. Culturally tailored intervention for rural African Americans with type 2 diabetes. Diabetes Educ. 2008;34(5):854–65. 18832290 10.1177/0145721708323642 PMC3622474

[29] Shehata AAM. Evaluating the effect of health education program on outcomes of type I diabetic patients: a randomized controlled study. Saudi J Biol Sci. 2020;27(11):2869–75. 33100841 10.1016/j.sjbs.2020.09.018 PMC7569108

[30] Andreae SJ, Andreae LJ, Cherrington A, Richman J, Safford M. Peer coach delivered storytelling program for diabetes medication adherence: intervention development and process outcomes. Contemp Clin Trials Commun. 2020;20:100653. 33024882 10.1016/j.conctc.2020.100653 PMC7527718

[31] Naranjo D, D. Schwartz D, M. Delamater A: Diabetes in ethnically diverse youth: disparate burden and intervention approaches. Curr Diabet Rev. 2015,;11(4):251–260. 10.2174/1573399811666150421115846 25901501

[32] Lado JJ, Lipman TH. Racial and ethnic disparities in the incidence, treatment, and outcomes of youth with type 1 diabetes. Endocrinol Metab Clin North Am. 2016;45(2):453–61. 27241975 10.1016/j.ecl.2016.01.002

[33] Silverstein J, Klingensmith G, Copeland K, Plotnick L, Kaufman F, Laffel L, et al. Care of children and adolescents with type 1 diabetes: a statement of the American Diabetes Association. Diabetes Care. 2005;28(1):186–212. 15616254 10.2337/diacare.28.1.186

[34] Zoffmann V, Lauritzen T. Guided self-determination improves life skills with type 1 diabetes and A1C in randomized controlled trial. Patient Educ Couns. 2006;64(1–3):78–86. 16720089 10.1016/j.pec.2005.11.017

[35] Rosenbek Minet L, Wagner L, Lønvig E, Hjelmborg J, Henriksen J. The effect of motivational interviewing on glycaemic control and perceived competence of diabetes self-management in patients with type 1 and type 2 diabetes mellitus after attending a group education programme: a randomised controlled trial. Diabetologia. 2011;54:1620–9. 21455729 10.1007/s00125-011-2120-x

[36] Hermanns N, Kulzer B, Ehrmann D, Bergis-Jurgan N, Haak T. The effect of a diabetes education programme (PRIMAS) for people with type 1 diabetes: results of a randomized trial. Diabetes Res Clin Pract. 2013;102(3):149–57. 24210673 10.1016/j.diabres.2013.10.009

[37] SPIRIT 2013 Checklist [https://view.officeapps.live.com/op/view.aspx?src=https%3A%2F%2Fwww.spirit-statement.org%2Fwp-content%2Fuploads%2F2013%2F08%2FSPIRIT-Checklist-download-8Jan13.doc&wdOrigin=BROWSELINK]

[38] Glasgow RE, Vogt TM, Boles SM. Evaluating the public health impact of health promotion interventions: the RE-AIM framework. Am J Public Health. 1999;89(9):1322–7. 10474547 10.2105/ajph.89.9.1322 PMC1508772

[39] Fisher L, Hessler D, Naranjo D, Polonsky W. AASAP: a program to increase recruitment and retention in clinical trials. Patient Educ Couns. 2012;86(3):372–7. 21831557 10.1016/j.pec.2011.07.002 PMC3219807

[40] Miller WR, Rollnick S. Motivational interviewing: Helping people change. Guilford press; 2012.

[41] Funnell MM, Anderson RM. Empowerment and self-management of diabetes. Clin Diabetes. 2004;22(3):123–8.

[42] Funnell MM, Anderson RM, Arnold MS, Barr PA, Donnelly M, Johnson PD, et al. Empowerment: an idea whose time has come in diabetes education. Diabetes Educ. 1991;17(1):37–41. 1986902 10.1177/014572179101700108

[43] Funnell MM, Nwankwo R, Gillard ML, Anderson RM, Tang TS. Implementing an empowerment-based diabetes self-management education program. Diabetes Educ. 2005;31(1):53–61. 15779247 10.1177/0145721704273166

[44] Ying X, Robinson KA, Ehrhardt S. Re-evaluating the role of pilot trials in informing effect and sample size estimates for full-scale trials: a meta-epidemiological study. BMJ Evid Based Med. 2023;28(6):383. 37491141 10.1136/bmjebm-2023-112358

[45] Gaglio B, Shoup JA, Glasgow RE. The re-aim framework: a systematic review of use over time. Am J Public Health. 2013;103(6):e38–46. 23597377 10.2105/AJPH.2013.301299 PMC3698732

[46] Glasgow RE, Riley WT. Pragmatic measures: what they are and why we need them. Am J Prev Med. 2013;45(2):237–43. 23867032 10.1016/j.amepre.2013.03.010

[47] Measuring Fidelity [https://www.jbassoc.com/resource/measuring-implementation-fidelity-2/]

[48] Phillips KA, Epstein DH, Mezghanni M, Vahabzadeh M, Reamer D, Agage D, et al. Smartphone delivery of mobile HIV risk reduction education. AIDS Res Treat. 2013;2013(1):231956. 24159383 10.1155/2013/231956 PMC3789326

[49] Fisher L, Polonsky WH, Hessler DM, Masharani U, Blumer I, Peters AL, et al. Understanding the sources of diabetes distress in adults with type 1 diabetes. J Diabetes Complications. 2015;29(4):572–7. 25765489 10.1016/j.jdiacomp.2015.01.012 PMC4414881

[50] Polonsky WH, Fisher L, Earles J, Dudl RJ, Lees J, Mullan J, et al. Assessing psychosocial distress in diabetes: development of the diabetes distress scale. Diabetes Care. 2005;28(3):626–31. 15735199 10.2337/diacare.28.3.626

[51] Schmitt A, Reimer A, Hermanns N, Huber J, Ehrmann D, Schall S, et al. Assessing diabetes self-management with the diabetes self-management questionnaire (DSMQ) can help analyse behavioural problems related to reduced glycaemic control. PLoS One. 2016;11(3):e0150774. 26938980 10.1371/journal.pone.0150774 PMC4777391

[52] T1-DDS: For adults with type 1 diabetes [https://diabetesdistress.org/dd-assess-score-2/]

[53] Totton N, Lin J, Julious S, Chowdhury M, Brand A. A review of sample sizes for UK pilot and feasibility studies on the ISRCTN registry from 2013 to 2020. Pilot Feasibility Stud. 2023;9(1):188. 37990337 10.1186/s40814-023-01416-w PMC10662929

[54] Lancaster GA, Dodd S, Williamson PR. Design and analysis of pilot studies: recommendations for good practice. J Eval Clin Pract. 2004;10(2):307–12. 15189396 10.1111/j..2002.384.doc.x

[55] Julious SA. Sample size of 12 per group rule of thumb for a pilot study. Pharm Stat. 2005;4(4):287–91.

[56] Viechtbauer W, Smits L, Kotz D, Budé L, Spigt M, Serroyen J, et al. A simple formula for the calculation of sample size in pilot studies. J Clin Epidemiol. 2015;68(11):1375–9. 26146089 10.1016/j.jclinepi.2015.04.014

[57] Carpenter J, Kenward M. Missing data in randomised controlled trials: a practical guide. In: Health Technology Assessment Methodology Programme. edn. Birmingham; 2007:199.

[58] Kvale S. Doing interviews. Sage Publications Ltd; 2012.

[59] NVIVO. QSR International. 2018. Retrieved September 20, 2018 from https://www.qsrinternational.com/nvivo/what-is-nvivo.

[60] Gibbs GR. Analyzing qualitative data. 2nd edn. SAGE Publications, Ltd; 2018.

