# Supplementary material for: Feasibility and signals of efficacy of the Type 1 Diabetes Education and Support (T1DES) intervention to improve diabetes distress and glycemic levels among Black young adults with type 1 diabetes compared to standard diabetes education: study protocol for a randomized pilot trial
Source: Pilot Feasibility Stud. 2026 Apr 1;12:65. doi: 10.1186/s40814-026-01776-z (PMC13169934; doi:10.1186/s40814-026-01776-z)
Supplement: Supplementary file 1 — Supplementary material 1. Appendix Consent forms. [file 40814_2026_1776_MOESM1_ESM.pdf]

KAISER FOUNDATION HEALTH PLAN OF GEORGIA, INC.

CONSENT TO PARTICIPATE IN A MEDICAL RESEARCH STUDY AND AUTHORIZATION TO USE AND DISCLOSE PROTECTED HEALTH INFORMATION FOR RESEARCH PURPOSES

STUDY TITLE: Evaluating the feasibility of Type 1 Diabetes Education and Support (T1DES) intervention to improve diabetes distress among Black young adults

STUDY SPONSOR: National Institute of Diabetes and Digestive and Kidney Diseases (**NIDDK) and Hemsley Charitable Trust**

STUDY INVESTIGATOR: Teaniese Davis, PhD, MPH and Priyathama Vellanki, MD

STUDY LOCATION: Atlanta, Georgia

STUDY CONTACT PHONE NUMBER: 470-834-9071

Key Information Summary

T1DES is a multisite study including participants from Kaiser Permanente Georgia and Grady Health System. The purpose of this research study is to compare the effects of behavioral intervention with traditional diabetes education on diabetes health outcomes among black young adults ages 18-30 years of age. This is being led by people at Kaiser Permanente Georgia, Emory, and Grady Health System. The behavioral intervention is a diabetes management intervention to improve diabetes distress. Diabetes distress is the negative emotional effect of living with diabetes. We are conducting this study because recent research has demonstrated racial and ethnic-based disparities among young adults with Type 1 Diabetes, with Black youth having poorer control of their diabetes. Additionally, Type 1 diabetes management interventions have been predominantly geared to white populations.

You are being asked to consent to participate in this research study. Your participation is completely voluntary. As part of this study, you will be assigned to one of two groups. One group will receive traditional diabetes education; the other will receive a behavioral intervention.

The study will last approximately 6 months. As part of this study, each group will be asked to participate in 5 information session over the course of the first three month; the first of which will be in person at a Kaiser Permanente Georgia, Emory, or Grady offices; or community partner sites in metropolitan Atlanta. Prior to or during this first session you will be asked to complete a survey about your diabetes. At the first session study staff will administer a point of care (POC) A1c test.. This is a finger stick to obtain your Hemoglobin A1c value. Once that is complete, the presentation of the content for the first information session will follow. Information sessions 2-5 will be scheduled to occur between baseline and 3 months after that and will be conducted virtually via phone or an online meeting platform like zoom or teams. Any virtual and in person meetings may be recorded and may be transcribed by an approved vendor for study purposes.

We will also ask you to recomplete the survey and get a new POC A1C in person at 3 months and 6 months. At the end of study, an exit focus group will be conducted. The focus group may also be recorded and transcribed by an approved vendor for study purposes.

Approved Date: March 13, 2024
Kaiser Permanente - Interregional IRB (KPiIRB)

This study involves minimal risk, the main ones resulting from the finger stick and the possibility of loss of your privacy and confidentiality. To minimize this risks, trained staff will be conducting the finger stick. We will also store all data in password protected devices and paper documents in locked cabinets.

It is not possible to predict whether you will receive any direct benefit as a result of your participation in this study., however information collected will help us improve our behavioral intervention which future patients with diabetes might benefit from. Please note the T1DES study team does not change who is managing your diabetes care and does not change your diabetes management plan. You should continue your care with your current provider.

Researchers at Kaiser Permanente in Georgia, Emory, and Grady Health System are conducting a research study. To decide whether you want to be part of this research, you should understand the risks and benefits in order to make an informed decision. You have the right to know what the purpose of the study is, how participants are selected, what procedures will be used, what the potential risks and benefits and possible alternative treatments are, what is expected of you as a study participant, and to inform you of how your personal health information may be used or given to others during the study and after the study is finished. This process is called "informed consent." This consent form gives information about the research study, which the study staff will discuss with you.

This consent form may contain words or phrases that you do not understand. Please ask the study doctor or the study staff to explain any words or information that you do not clearly understand. If you decide that you not want to participant today, you may still be able to participate at a later date. You may take home an unsigned copy of this consent form to think about or discuss the study with family or friends before making your decision. Once you are satisfied that you understand the study, you will be asked to sign and date this consent form if you choose to participate. You will be given a copy of the signed and dated consent form.

Who is funding this study?
The research costs of this study are being paid by the study sponsors, the National Institute of Diabetes and Digestive and Kidney Diseases and Helmsley Charitable Trust. Kaiser Permanente, Emory, and Grady Health System will be reimbursed for the time and resources used in conducting this study on behalf of the sponsors.

What is the purpose of this study?
The purpose of this study is to assess the effectiveness of an intervention to enhance diabetes management strategies among Black young adults with Type 1 Diabetes and compare it with traditional diabetes education.

Why am I being asked to take part in this study?
You are being asked to take part in this research study because you:

- identify as Black or African American
- diagnosed with Type 1 Diabetes
- are 18-30 years of age
- have a recent Lab A1c >7.5 or a POC A1C >7.5

How many people will take part in this study?
We will enroll 80 individuals (40 from Grady Health System and 40 from Kaiser Permanente Georgia). 40 will be assigned to each of the two study groups.

How long will I be in this study?

The study will last 6 months.

What will happen if I take part in this study?
If you agree to take part in this study and sign this consent form, the following things will happen:

You will be randomized, much like the flip of a coin, into one of two groups:

- Group 1 - Diabetes education group: This group will focus on diabetes education and management strategies, including discussing how you manage your diabetes equipment.
- Group 2 - Behavioral intervention group: This group will focus on strategies to improve diabetes distress.

The randomization process is used to make sure study results are not influenced by the selection of participants in one group as compared to another. Please note the T1DES study team does not change who is managing your diabetes care and does not change your diabetes management plan. You should continue your care with your current providers.

- Before Day 1: You will have an opportunity to complete the survey before you arrive using a link. The survey will ask you questions about your diabetes management and diabetes distress. This will take approximately 15-30 minutes.

- Day 1: For those that have not completed the survey, you will complete the survey at this appointment. You will complete a POC A1C. The POC A1c is a finger stick to obtain your A1c value. This initial session will last about 4 hours.

- Then you will complete the remaining sessions 2-5 over the next 3 months of the study. Depending on which group you are assigned to, these follow up sessions will last between 30 to 60 minutes.
    - For those individuals assigned to the diabetes education group, you will be asked to complete tracking logs for your diabetes data and nutrition prior to these sessions and share it with your facilitator.
    - For those individuals assigned to the behavioral intervention, you will be asked to complete worksheets before each session and may be asked to share during the session.

- Month 3: At the 3-month time point you will complete a POC A1C in-person and a survey.

- Month 6: At the 6-month time point you will complete ae POC A1C in-person and a survey.

- At the end of the study (month 6) an exit focus group will also be conducted. The focus group will also last about an hour.

- Please note that any of the study sessions and focus group including virtual and in person meetings may be audio and video recorded and transcribed by an approved vendor for study purposes. Results from each session will be transferred into a written report, but no names will be used in the document. We need the audio tape recording and notes to have an accurate record of what is discussed during each session.

Contact Information: We will collect your personal contact information to follow-up with you. We may ask you to provide an alternative contact number or person - this is completely voluntary. We will only contact this alternate contact in case we are unable to reach you via the personal contact information you provide (disconnected line, email bounces back, no answer after 3 attempts, etc.). We will not inform the person you list as the alternate contact any details about the nature of the research study; we will just inform them that we are from one of the participating study locations and inquire if there is another way to contact you.

Text Messages: As a participant in this study, you will also receive study communications via an online text messaging platform called Twilio. These communications will remind you of upcoming sessions, study alerts,

measurement, and follow-up appointments. It will also be used for participation satisfaction survey responses throughout the program. We will use an approved third-party system that will enable direct communication between you and the T1DES study team.

Follow-up Communication: Communications relevant to the study will be done over email, mail, phone calls, and text messages. We may also use GroupMe for study communications.

Communicating with the Research Team by Text

The research team will contact you by phone, email or text messages, depending on your preference. There is no way to protect ("encrypt") information in the messages sent by text. This means that information you send or receive by text message could be looked at by someone who was not supposed to see it, or by your mobile/cell phone provider or company. Therefore, when text messages are sent, there may be risks related to your privacy. We would like to use text messages to remind you about visits, send you links to surveys and give you other information about the study. We will not send you test results by text. Therefore, when text messages are sent, there may be risks related to your privacy.

Please indicate whether you agree to receive text messages from the research team: *Please check one.*

- ☐ Yes, I agree to receive text messages from the research team.
- ☐ No, I do not agree to receive text messages from the research team

Will the information collected be used in future research?

Your information will not be stored or used for future research**.**

What are the potential risks, side effects and discomforts of being in this study?
Risks related to the finger stick:
There is a minor risk associated with this project in that you may experience slight pain when we pierce the skin on your finger to conduct the POC A1c; however the puncture and blood collecting equipment are part of the commercially available systems that have been approved by the FDA.

Drawing blood from a finger stick may, in rare cases- cause discomfort, bruising, prolonged bleeding and infection at the site of puncture. To minimize risk, we will swab the site of puncture with alcohol to disinfect the area, use disposable lancet and capillary tubes to collect blood and apply pressure to the puncture site following the blood draw to minimize bruising. We will cover the puncture with an appropriate dressing and provide you with information on how to monitor for signs of infection.

Survey: Some of these questions may seem very personal or embarrassing. They may upset you. You may refuse to answer any of the questions that you do not wish to answer. If the questions make you very upset, we will help you to find a counselor.

Other Risks: There may be other risks such as exposure to COVID-19 or risks that we do not know at this time. In the event that any of these unforeseeable risks happens, we urge you to tell us about any unusual symptoms. Tell us even if you feel these symptoms are mild or do not bother you.

<u>Privacy Risks</u>
There is a small chance that being in this study may involve a loss of privacy. State and federal laws require Kaiser Permanente to keep your health information private and safe. In this study, your information is going outside Kaiser Permanente to study team members not affiliated with Kaiser Permanente, Although Kaiser Permanente requires these outside researchers to keep your information private and safe, the laws that protect your information may not apply. Therefore, Kaiser Permanente cannot guarantee that your information will be protected once it is sent outside

Approved Date: March 13, 2024
Kaiser Permanente - Interregional IRB (KPiIRB)

of Kaiser Permanente.

Are there any benefits to being in this study?

It is not possible to predict whether or not you will receive any direct benefit as a result of your participation in this study. However, it is hoped that the results of this study may benefit other patients in the future.

What are my choices if I do not want to be in this study?

Participation in this study is completely voluntary. You are free to refuse to participate in this study. Your decision on whether or not to participate in the study will not affect your medical care. If you decide to participate, you are free to change your mind and discontinue participation at any time without any effect on your medical care or eligibility for future care or membership in Kaiser Permanente, Emory, or Grady Health System.

Will there be any costs to me to take part in this study?

There will be no cost to you to participate in this study.

- Grady Health System will provide a transportation voucher for each study visit, not to exceed $20.
- For all participants we will cover the cost of parking.

Will I be paid to take part in this study?
You will be given a reloadable gift card and paid only for activities you complete.

**Study Appointments**	**Compensation**
Baseline, (1st Assessment and POC A1C (in person)	$50
Session 1 Attendance (in person)	$50
Sessions 2-5 Attendance (virtual)	$25 per session (up to $100)
3 Month, 2nd Assessment including POC A1C (in person)	$50
6 Month, 3rd Assessment including POC A1C (in person)	$50
Exit Focus Group(virtual or in-person)	$50
Maximum Total	$350

If you receive more than $300 in compensation for your study activities:

You will be asked to fill out a tax form, including your Social Security or Taxpayer Identification Number, in order to be reimbursed, depending on the amount and method of payment. Some payment methods involve mail coming to your house, which may be seen by others in your household. You can decline payment if you are concerned about confidentiality, or you can talk to the study team to see if there are other payment options.

What will happen if I am injured during the study?

We will give you emergency care if you are injured by this research. However, **Grady Health System**, **Kaiser Foundation Health Plan, Inc**., **Kaiser Foundation Hospitals**, **The Permanente Medical Group, Inc**., or the **staff conducting the study or any of the participating research sites** have not set aside funds to pay for this care or to compensate you if a mishap occurs. If you believe you have been injured by this research, you should contact Dr. Vellanki, Grady Health System (Phone 404-778-1687) or Dr. Davis, Kaiser Permanente (Phone 404-337-3646).

Your consent to participate in this research study does not take away any legal rights which you may have in the case of negligence or legal fault of anyone who is involved with this study.

Will my information be kept confidential?
Efforts will be made to keep your personal information confidential. However, your personal information may be disclosed if required by law.

To help keep information about you confidential, we have received a Certificate of Confidentiality from the federal government. The Certificate protects against the involuntary release of information about you collected during the course of this study. The researchers involved in this study cannot be forced to disclose your identity or any information about you collected in this study in any legal proceedings at the federal, state, or local level. However, you or the researcher may choose to voluntarily disclose the protected information under certain circumstances. For example, we may disclose medical information in cases of medical necessity or take steps (including notifying authorities) to protect you or someone else from serious harm, including child abuse. Additionally, if you request the release of information about you in writing (through, for example, a written request to release medical records to an insurance company), the Certificate does not protect against that voluntary disclosure. This certificate does not prevent the researchers from releasing information about you to prevent serious harm to you or someone else. Moreover, federal agencies may review our records under limited circumstances, such as a Department of Health and Human Services request for information for an audit or program evaluation.

To the extent permitted by law and by signing this consent form, you allow access for the following representatives to inspect your research and clinical records without removal of identifying information, such as your name, initials, date of birth, sex, and race, to make sure that the information is correct and to evaluate the conduct of the study.

- The sponsors of this study, **NIDDK/NIH**, **Helmsley Charitable Trust** and/or their authorized representatives;
- The U.S. Food and Drug Administration (FDA); the Department of Health and Human Services (DHHS); or other governmental regulatory agencies [in the US and other countries] involved in keeping research safe for people;
- Kaiser Permanente Interregional Institutional Review Board (a formal committee that reviews research studies to protect the rights and welfare of participants);
- Representatives of Kaiser Permanente, Emory, and Grady Health System

Because of the need to allow access to your information to these parties, absolute confidentiality cannot be guaranteed.

All study records will identify you through a unique code number. The study investigator will ensure that the link between your name and these code numbers will never be released to those outside of the research team. All coded records will be kept confidential and stored in a secured area and electronic documents linking your name to your code will be password protected.

Because of the need to allow access to your cellphone number to Twilio and GroupMe, absolute confidentiality cannot be guaranteed.

If you decide to participate in this study, you will also be giving consent for the medical research investigator or his/her assistants to review your medical records as may be necessary for this study.

No results from the study will be placed in your medical record..

Follow up*: Please check here if we have permission to contact you about follow-up studies.*

☐ In the future, we may contact you to offer you the opportunity to take part in other studies.

How will my health information be used and disclosed in this study?
The privacy of your health information is important to us. As part of this study, we will get your protected health

Approved Date: March 13, 2024
Kaiser Permanente - Interregional IRB (KPiIRB)

information (PHI) from health care entities who are covered by the Health Insurance Portability and Accountability Act and regulations (HIPAA). Because the health care entities are covered by HIPAA, we must have your authorization to get your PHI from them. However, once we get your PHI from the health care entities, it changes from PHI to individually identifiable information (IIHI) and is no longer covered by HIPAA. We will put your IIHI in a separate research record that is not a part of your medical record. IIHI placed in the separate research record is not covered by HIPAA.

No Provision of Treatment
There is no research-related treatment involved in this study. You may receive any non-research related treatment whether or not you sign this form.

IIHI that Will be Used/Disclosed:

The IIHI that we will use or disclosed for this study includes:
- Medical information about you including your medical history and present/past medications.
- Results of exams, procedures and tests you have before and during the study.
- Laboratory test results.

Purposes for Which Your IIHI Will be Used/Disclosed:
- To conduct this research study
- To evaluate the safety and effectiveness of the intervention being studied and ensure integrity of the data
- To provide study-related treatment
- To conduct healthcare operations
- To ensure compliance with state and federal regulations and provide oversight of the study
- To determine your health, vital status or contact information should you be unreachable during the study
- For the administration and payment of any costs relating to subject injury from the study

Use and Disclosure of Your IIHI That is Required by Law:
We will use and disclose your IIHI when we are required to do so by law. This includes laws that require us to report child abuse or abuse of elderly or disabled adults.

Authorization to Use IIHI is Required to Participate:
By signing this form, you give us permission to use and disclose your IIHI for this research study.

People Who will Use/Disclose Your IIHI:

- The Principal Investigator and the research staff
- The sponsor of the research, its agents, study monitors and contractors including laboratories if applicable
- Institutional Review Boards (people who provide ethical review of research)
- Other Emory offices and persons who watch over the safety, effectiveness and conduct of the research
- Government agencies that regulate the research as applicable to this study (e.g. regulatory agencies within and outside the United States such as the Office for Human Research Protections, Food and Drug Administration and Veterans Administration)

Your cellphone number will be entered into approved, external database platforms by you and the research team for the purpose of study communication. Cellphone information will be shared with following:
- Twilio
- GroupMe

In certain cases where a researcher moves to a different institution, your IIHI may be disclosed to that new institution and their oversight offices. The IIHI will be disclosed in a secure manner and under a legal agreement signed by both institutions to ensure it continues to be used under the terms of this consent and authorization.

Expiration of Your Authorization
Your HIPAA authorization will expire once no more PHI is needed from your medical records for this study.

Revoking Your Authorization
If you sign this form, at any time later you may revoke (take back) your permission to use your IIHI. If you want to do this, you must contact the study team at:
Priyathama Vellanki
Glenn Memorial Building
Suite 200
69 Jesse Hill Dr SE
Atlanta, GA 30303

At that point, we will stop collecting your IIHI. We may use or disclose the IIHI already collected so we can follow the law, protect your safety, make sure that the study was done properly and the data are correct. If you revoke your authorization you will not be able to stay in the study.

Other Items You Should Know about Your Privacy
Not all people and entities are covered by the Privacy Rules. HIPAA only applies to health care providers, health care payers, and health care clearinghouses. HIPAA does not apply to the research records for this study because the study does not include treatment that is billed to insurers or government benefit programs. Your information collected for this study may be disclosed to others without your permission. The researchers, Sponsor, and people and companies working on this study are not covered by the Privacy Rules. They will only use and disclose your information as described in this Consent and Authorization.

To maintain the integrity of this research study, you generally will not have access to your IIHI related to this research until the study is complete. When the study ends, and at your request, you generally will only have access to your IIHI that we maintain in a designated record set. A designated record set is data that includes medical information or billing records that your health care providers use to make decisions about you. You will not have a right of access to IIHI kept in a separate research record used only for research purposes. If it is necessary for your health care, your health information will be provided to your doctor.

We may remove identifying information from your IIHI. Information without identifiers is not subject to HIPAA and may be used or disclosed with other people or organizations for purposes besides this study.

Am I required to be in this study?
Participation in this study is completely voluntary. You are free to refuse to participate in this study. Your decision whether or not to participate in the study will not affect your medical care. If you decide to participate, you are free to change your mind and discontinue participation at any time without any effect on your medical care or eligibility for future care or membership in KFHP, Emory, or Grady Health System

If you leave the research, information collected while you were in the study will not be removed from our records.

Will I receive results from the POC A1c test in this study?

We do not intend to provide POC A1c test results, but you are free to ask for your results when the test is being administered.

Will I receive new information about the study while participating?
During the course of the study, you will be informed of any important new findings (either good or bad) such as changes in the risks or benefits resulting from participation in the research or new alternatives that might change your mind about your continued participation in the study. You may be asked to sign a new consent form if additional risks are found.

Where can I get more information?
A description of this clinical trial will be available on http://www.ClinicalTrials.gov, as required by U.S. Law. This Web site will not include information that can identify you. At most, the web site will include a summary of the results. You can search this Web site at any time.

What if I have any questions or problems?
In case of study-related questions, problems or injuries, you can call the investigators responsible for the study.
Teaniese Davis, PhD, MPH at 404-337-3646
Priyathama Vellanki, MD at 404-778-1687

If you are a patient receiving care from the Grady Health System and have a question about your rights, you may contact the Office of Research Administration at research@gmh.edu

Questions about your rights as a study participant, comments or complaints about the study may be presented to the Kaiser Permanente Interregional Institutional Review Board:

Phone: (951) 739-6781
email: KPINTERREGIONALIRB@kp.org

CONSENT AND AUTHORIZATION TO BE IN THE STUDY:

I have read (or someone has read to me) the above and am satisfied with my understanding of the study, its possible benefits, risks and alternatives. I have been given an opportunity to ask questions and my questions have been answered to my satisfaction. By signing this form, I authorize Kaiser Permanente, Emory, and Grady Health System researchers to use and disclose my PHI for the purpose of this research study. I will be given a copy of this consent form, which includes the Authorization to Use and Disclose Protected Health Information for Research Purposes.
BY SIGNING BELOW, I WILLINGLY AGREE TO PARTICIPATE IN THE RESEARCH STUDY AND AUTHORIZE THE USE AND DISCLOSURE OF MY PHI AS DESCRIBED IN THIS FORM.

____
Printed Name of Participant

____________________________________ _______________
Signature of Participant Date

I have explained the nature and purpose, the potential benefits and possible risks associated with participation in this research study to the participant. I have answered any questions that have been raised and have witnessed the above signatures.

____
Printed Name of Person Obtaining Consent

____ __________________
Signature of Person Obtaining Consent Date

Approved Date: March 13, 2024
Kaiser Permanente - Interregional IRB (KPiIRB)

KAISER FOUNDATION HEALTH PLAN OF GEORGIA, INC.

CONSENT TO PARTICIPATE IN A MEDICAL RESEARCH STUDY AND AUTHORIZATION TO USE AND DISCLOSE PROTECTED HEALTH INFORMATION FOR RESEARCH PURPOSES

STUDY TITLE: Evaluating the feasibility of Type 1 Diabetes Education and Support (T1DES) intervention to improve diabetes distress among Black young adults

STUDY SPONSOR: National Institute of Diabetes and Digestive and Kidney Diseases (**NIDDK)**

STUDY INVESTIGATOR: Teaniese Davis, PhD, MPH

STUDY LOCATION: Atlanta, Georgia

STUDY CONTACT PHONE NUMBER: 470-834-9071

Key Information Summary

The purpose of this research study is to compare the effects of behavioral intervention with traditional diabetes education on diabetes health outcomes among black young adults ages 18-30 years of age. The behavioral intervention is a diabetes management intervention to improve diabetes distress. Diabetes distress is the negative emotional effect of living with diabetes. We are conducting this study because recent research has demonstrated racial and ethnic-based disparities among young adults with Type 1 Diabetes, with Black youth having poorer control of their diabetes. Additionally, Type 1 diabetes management interventions have been predominantly geared to white populations.

You are being asked to consent to participate in this research study. Your participation is completely voluntary. As part of this study, you will be assigned to one of two groups. One group will receive traditional diabetes education; the other will receive a behavioral intervention.

The study will last approximately 6 months. As part of this study, each group will be asked to participate in 5 information session over the course of the first three month; the first of which will be in person at one of the Kaiser Permanente Georgia offices or community partner sites in metropolitan Atlanta. Prior to or during this first session you will be asked to complete a survey about your diabetes. And study staff will administer a point of care (POC) A1c test for individuals without one. This is a finger stick to obtain your Hemoglobin A1c value. Once that is complete, the presentation of the content for the first information session will follow. Information sessions 2-5 will be scheduled to occur between baseline and 3 months after that and will be conducted virtually via phone or an online meeting platform like zoom or teams. Any virtual and in person meetings may be recorded and may be transcribed by a KP approved vendor for study purposes.

We will also ask you to recomplete the survey and get a new POC A1C in person at 3 months and 6 months. At the end of study, an exit focus group will be conducted. The focus group may also be recorded and transcribed by a KP approved vendor for study purposes.

This study involves minimal risk, the main ones resulting from the finger stick and the possibility of loss of your privacy and confidentiality. To minimize this risks, trained staff will be conducting the finger stick. We will also store all data in password protected devices and paper documents in locked cabinets.

It is not possible to predict whether you will receive any direct benefit as a result of your participation in this study., however information collected will help us improve our behavioral intervention which future patients with diabetes

Approved Date: July 22, 2024
Kaiser Permanente - Interregional IRB (KPiIRB)

might benefit from. Please note the T1DES study team does not change who is managing your diabetes care and does not change your diabetes management plan. You should continue your care with your current provider.

Researchers at Kaiser Permanente in Georgia are conducting a research study. To decide whether you want to be part of this research, you should understand the risks and benefits in order to make an informed decision. You have the right to know what the purpose of the study is, how participants are selected, what procedures will be used, what the potential risks and benefits and possible alternative treatments are, what is expected of you as a study participant, and to inform you of how your personal health information may be used or given to others during the study and after the study is finished. This process is called "informed consent." This consent form gives information about the research study, which the study staff will discuss with you.

This consent form may contain words or phrases that you do not understand. Please ask the study doctor or the study staff to explain any words or information that you do not clearly understand. If you decide that you not want to participant today, you may still be able to participate at a later date. You may take home an unsigned copy of this consent form to think about or discuss the study with family or friends before making your decision. Once you are satisfied that you understand the study, you will be asked to sign and date this consent form if you choose to participate. You will be given a copy of the signed and dated consent form.

Who is funding this study?
The research costs of this study are being paid by the study sponsor, the National Institute of Diabetes and Digestive and Kidney Diseases. Kaiser Permanente will be reimbursed for the time and resources used in conducting this study on behalf of the sponsor.

What is the purpose of this study?
The purpose of this study is to assess the effectiveness of an intervention to enhance diabetes management strategies among Black young adults with Type 1 Diabetes and compare it with traditional diabetes education.

Why am I being asked to take part in this study?
You are being asked to take part in this research study because you have been

- identify as Black or African American
- diagnosed with Type 1 Diabetes
- are 18-30 years of age
- have a recent Lab A1c >7.5 or a POC A1C >7.5

How many people will take part in this study?
We will enroll 40 individuals. 20 will be assigned to each of the two groups.

How long will I be in this study?

The study will last 6 months.

What will happen if I take part in this study?
If you agree to take part in this study and sign this consent form, the following things will happen:

You will be randomized, much like the flip of a coin, into one of two groups:

- Group 1 - Diabetes education group: This group will focus on diabetes education and management strategies, including discussing how you manage your diabetes equipment.
- Group 2 - Behavioral intervention group: This group will focus on strategies to improve diabetes distress.

Approved Date: July 22, 2024
Kaiser Permanente - Interregional IRB (KPiIRB)

The randomization process is used to make sure study results are not influenced by the selection of participants in one group as compared to another. Please note the T1DES study team does not change who is managing your diabetes care and does not change your diabetes management plan. You should continue your care with your current providers.

- Day 0: Prior to coming in for your first session we give you the opportunity to complete the survey virtually.

- Day 1: For those that have not completed the survey, you will complete the survey at this appointment. The survey will ask you questions about your diabetes management and diabetes distress. You will complete a Point of Care A1C. The POC A1c is a finger stick to obtain your A1c value. This initial session will last about 4 hours.

- Then you will complete the remaining sessions 2-5 over the next 3 months of the study. Depending on which group you are assigned to, these follow up sessions will last between 30 to 60 minutes.
    - For those individuals assigned to the diabetes education group, you will be asked to complete tracking logs for your diabetes data and nutrition prior to these sessions and share it with your facilitator.
    - For those individuals assigned to the behavioral intervention, you will be asked to complete worksheets before each session and may be asked to share during the session.

- Month 3: At the 3-month time point you will complete a POC A1C in-person and a survey.
- Month 6: At the 6-month time point you will complete ae POC A1C in-person and a survey.

- At the end of the study (month 6) an exit focus group will also be conducted. The focus group will also last about an hour.

- Please note that any of the study sessions and focus group including virtual and in person meetings may be audio and video recorded and transcribed by a KP approved vendor for study purposes. Results from each session will be transferred into a written report, but no names will be used in the document. We need the audio tape recording and notes to have an accurate record of what is discussed during each session.

Contact Information: We will collect your personal contact information to follow-up with you. We may ask you to provide and alternative contact number or person - this is completely voluntary. We will only contact this alternate contact in case we are unable to reach you via the personal contact information you provide (disconnected line, email bounces back, no answer after 3 attempts, etc.). We will not inform the person you list as the alternate contact any details about the nature of the research study; we will just inform them that we are from the Kaiser Permanente Center for Research and Evaluation and inquire if there another way to contact you.

Text Messages: As a participant in this study, you will also receive study communications via an online text messaging platform called Twilio. These communications will remind you of upcoming sessions, study alerts, measurement, and follow-up appointments. It will also be used for participation satisfaction survey responses throughout the program. This is a secure, KP-approved third-party system that will enable direct communication between you and the T1DES study team.

Follow-up Communication: Communications relevant to the study will be done over email, mail, phone calls, and text messages. We may also use GroupMe for study communications.

Approved Date: July 22, 2024
Kaiser Permanente - Interregional IRB (KPiIRB)

Communicating with the Research Team by Text

The research team will contact you by phone, email or text messages, depending on your preference. There is no way to protect ("encrypt") information in the messages sent by text. This means that information you send or receive by text message could be looked at by someone who was not supposed to see it, or by your mobile/cell phone provider or company. Therefore, when text messages are sent, there may be risks related to your privacy. We would like to use text messages to remind you about visits, send you links to surveys and give you other information about the study. We will not send you test results by text. Therefore, when text messages are sent, there may be risks related to your privacy.

Please indicate whether you agree to receive text messages from the research team: *Please check one.*

- □ Yes, I agree to receive text messages from the research team.
- □ No, I do not agree to receive text messages from the research team

Will the information collected be used in future research?

Your information will not be stored or used for future research**.**

What are the potential risks, side effects and discomforts of being in this study?
Risks related to the finger stick:
There is a minor risk associated with this project in that you may experience slight pain when we pierce the skin on your finger to conduct the POC A1c; however the puncture and blood collecting equipment are part of the commercially available systems that have been approved by the FDA.

Drawing blood from a finger stick may, in rare cases- cause discomfort, bruising, prolonged bleeding and infection at the site of puncture. To minimize risk, we will swab the site of puncture with alcohol to disinfect the area, use disposable lancet and capillary tubes to collect blood and apply pressure to the puncture site following the blood draw to minimize bruising. We will cover the puncture with an appropriate dressing and provide you with information on how to monitor for signs of infection.

Survey: Some of these questions may seem very personal or embarrassing. They may upset you. You may refuse to answer any of the questions that you do not wish to answer. If the questions make you very upset, we will help you to find a counselor.

Other Risks: There may be other risks such as exposure to COVID-19 or risks that we do not know at this time. In the event that any of these unforeseeable risks happens, we urge you to tell us about any unusual symptoms. Tell us even if you feel these symptoms are mild or do not bother you.

Privacy Risks
There is a small chance that being in this study may involve a loss of privacy. State and federal laws require Kaiser Permanente to keep your health information private and safe. In this study, your information is going outside Kaiser Permanente to study team members not affiliated with Kaiser Permanente, Although Kaiser Permanente requires these outside researchers to keep your information private and safe, the laws that protect your information may not apply. Therefore, Kaiser Permanente cannot guarantee that your information will be protected once it is sent outside of Kaiser Permanente.

Are there any benefits to being in this study?

It is not possible to predict whether or not you will receive any direct benefit as a result of your participation in this study. However, it is hoped that the results of this study may benefit other patients in the future.

What are my choices if I do not want to be in this study?

Participation in this study is completely voluntary. You are free to refuse to participate in this study. Your decision on whether or not to participate in the study will not affect your medical care. If you decide to participate, you are free to change your mind and discontinue participation at any time without any effect on your medical care or eligibility for future care or membership in Kaiser Permanente.

Will there be any costs to me to take part in this study?

There will be no cost to you to participate in this study. All aspects of your standard medical care will continue to be provided to you according to the terms of your plan benefits described in your applicable plan Evidence of Coverage or Summary Plan Description, which may include copayments, coinsurance, and deductibles.

Will I be paid to take part in this study?
You will be given a reloadable gift card and paid only for activities you complete.

**Study Appointments**	**Compensation**
Baseline, (1st Assessment and POC A1C (in person)	$50
Session 1 Attendance (in person)	$50
Sessions 2-5 Attendance (virtual)	$25 per session (up to $100)
3 Month, 2nd Assessment including POC A1C (in person)	$50
6 Month, 3rd Assessment including POC A1C (in person)	$50
Exit Focus Group (virtual)	$50
Maximum Total	$350

We will also provide a transportation voucher for each study visit, not to exceed $20.
What will happen if I am injured during the study?

Any injury or condition experienced by a member of Kaiser Foundation Health Plan, Inc. as a result of being in this study will be treated and covered as described in your plan Evidence of Coverage or Summary Plan Description.

No free medical care or other form of compensation will be offered by Kaiser Foundation Health Plan, Inc., Kaiser Foundation Hospitals, The Permanente Medical Group, Inc., or the Kaiser Permanente staff conducting the study.

Your consent to participate in this research study does not take away any legal rights which you may have in the case of negligence or legal fault of anyone who is involved with this study.

Will my information be kept confidential?
Efforts will be made to keep your personal information confidential. However, your personal information may be disclosed if required by law.

To help keep information about you confidential, we have received a Certificate of Confidentiality from the federal government. The Certificate protects against the involuntary release of information about you collected during the course of this study. The researchers involved in this study cannot be forced to disclose your identity or any information about you collected in this study in any legal proceedings at the federal, state, or local level. However, you or the researcher may choose to voluntarily disclose the protected information under certain circumstances. For example, we may disclose medical information in cases of medical necessity or take steps (including notifying

authorities) to protect you or someone else from serious harm, including child abuse. Additionally, if you request the release of information about you in writing (through, for example, a written request to release medical records to an insurance company), the Certificate does not protect against that voluntary disclosure. This certificate does not prevent the researchers from releasing information about you to prevent serious harm to you or someone else. Moreover, federal agencies may review our records under limited circumstances, such as a Department of Health and Human Services request for information for an audit or program evaluation.

To the extent permitted by law and by signing this consent form, you allow access for the following representatives to inspect your research and clinical records without removal of identifying information, such as your name, initials, date of birth, sex, and race, to make sure that the information is correct and to evaluate the conduct of the study.

- The sponsor of this study, **NIDDK/NIH**, and/or its authorized representatives;
- The U.S. Food and Drug Administration (FDA); the Department of Health and Human Services (DHHS); or other governmental regulatory agencies [in the US and other countries] involved in keeping research safe for people;
- Kaiser Permanente Georgia Institutional Review Board (a formal committee that reviews research studies to protect the rights and welfare of participants);
- Representatives of Kaiser Permanente

Because of the need to allow access to your information to these parties, absolute confidentiality cannot be guaranteed.

All study records will identify you through a unique code number. The study investigator will ensure that the link between your name and these code numbers will never be released to those outside of the research team. All coded records will be kept confidential and stored in a secured area and electronic documents linking your name to your code will be password protected.

Because of the need to allow access to your cellphone number to Twilio and GroupMe, absolute confidentiality cannot be guaranteed.

If you decide to participate in this study, you will also be giving consent for the medical research investigator or his/her assistants to review your medical records as may be necessary for this study.

No results from the study will be placed in your medical record or shared with Kaiser Foundation Health Plan.

Follow up*: Please check here if we have permission to contact you about follow-up studies.*

☐ In the future, we may contact you to offer you the opportunity to take part in other studies.

How will my health information be used and disclosed in this study?
The Privacy Rule is a federal law designed to safeguard your Protected Health Information (PHI). Your PHI is individually identifiable information about you, some of which includes your physical or mental health, the receipt or provision of health care, or payment for that care. The Privacy Rule requires that researchers obtain your written authorization (approval) for us to use and disclose (release) your PHI. A disclosure of PHI means communicating that information to a person or research facility/company outside of KP Georgia.

Your PHI will only be used and disclosed as described in this authorization, except as otherwise required by law.

What is the purpose of the use or disclosure of my PHI?
Kaiser Permanente researchers will use your PHI, including your research and/or medical record, to conduct the study, monitor your health status, measure effects of drugs, and determine research results. In addition, others at Kaiser Permanente may also review your research or medical record, or both, to monitor the study. This will include

Approved Date: July 22, 2024
Kaiser Permanente - Interregional IRB (KPiIRB)

the Kaiser Permanente Interregional Institutional Review Board. Other external collaborators may also review your records.

What information will be used or disclosed?
To do this study, we will look at or collect information about you and your health. We will use and disclose your information electronically and/or via paper.

The following identifiable private information about you will be used and disclosed:

- Name
- Address, Phone Number, Email
- Dates, including birth date, admission date, discharge date
- Diabetes Status
- Information from study activities and POC A1c test results
- Demographic information: including but not limited to race, age, sex, education
- Medical record
- Digital and in-person Meeting Recordings and Transcriptions
- healthcare utilization from medical record
- medication usage from medical record
- Any electronic communications

Must I agree to this authorization to participate in the research?
Yes, in order to participate in this research study, you must agree to the uses and disclosures of your PHI as described in this authorization.

Who will use or disclose my PHI?
Kaiser Permanente researchers and the research team will use your PHI for the purposes of this study as described in the consent form.

If you sign this authorization, Kaiser Permanente researchers and the research team may use your PHI. They will use your study research record (the information that you provide in your answers to the questionnaires, information from your assessments and information from your medical record) to evaluate the study results.

Your cellphone number will be entered into approved, external database platforms by you and the Kaiser Permanente research team for the purpose of study communication. Cellphone information will be shared with following:

- Twilio
- GroupMe

Your PHI may also be sent to persons outside of KP Georgia assisting with this study or to others as required by law.

How will the confidentiality of my information be protected?
Kaiser Permanente is committed to protecting your personal health information. State and federal law also require Kaiser Permanente to maintain privacy and security of your information in this study. To protect the confidentiality of your information, we will keep your records secure through use of password protected files, encryption when sending study information over the internet, and storing paper documents in locked cabinets.

When will this authorization expire?
This agreement will expire at the end of the study.

Can I withdraw this authorization?
If at any time you want to withdraw from this agreement, you must notify us in writing:

Teaniese Davis, PhD, MPH
Kaiser Permanente -Center for Research and Evaluation
1375 Peachtree Street NE
Suite 380
Atlanta, GA 30309

After we receive your notification, we will continue to use only data that we have already obtained, unless we need to monitor your data for your safety.

What will happen to my PHI after it is disclosed?
The Kaiser Permanente research team will use and disclose your PHI only as described in this authorization. However, if someone receives your PHI from Kaiser Permanente and then discloses it again to someone else, it may no longer be protected by this authorization.

Can I see the information collected about me in this study?
You may not be allowed to review the information collected about you for this clinical trial until the study is over.

Am I required to be in this study?
Participation in this study is completely voluntary. You are free to refuse to participate in this study. Your decision whether or not to participate in the study will not affect your medical care. If you decide to participate, you are free to change your mind and discontinue participation at any time without any effect on your medical care or eligibility for future care or membership in KFHP.

If you leave the research, information collected while you were in the study will not be removed from our records.

Will I receive results from the POC A1c test in this study?

We do not intend to provide POC A1c test results, but you are free to ask for your results when the test is being administered.

Will I receive new information about the study while participating?
During the course of the study, you will be informed of any important new findings (either good or bad) such as changes in the risks or benefits resulting from participation in the research or new alternatives that might change your mind about your continued participation in the study. You may be asked to sign a new consent form if additional risks are found.

Where can I get more information?
A description of this clinical trial will be available on http://www.ClinicalTrials.gov, as required by U.S. Law. This Web site will not include information that can identify you. At most, the Web site will include a summary of the results. You can search this Web site at any time.

What if I have any questions or problems?
In case of study-related questions, problems or injuries, you can call the investigator responsible for the study within Kaiser Permanente in Georgia **Teaniese Davis, PhD, MPH** at 470-834-9071**.**

Questions about your rights as a study participant, comments or complaints about the study may be presented to the Kaiser Permanente Interregional Institutional Review Board:

Kaiser Permanente interregional IRB

Phone: (951) 739-6781
email: KPINTERREGIONALIRB@kp.org

CONSENT AND AUTHORIZATION TO BE IN THE STUDY:

I have read (or someone has read to me) the above and am satisfied with my understanding of the study, its possible benefits, risks and alternatives. I have been given an opportunity to ask questions and my questions have been answered to my satisfaction. By signing this form, I authorize Kaiser Permanente researchers to use and disclose my PHI for the purpose of this research study. I will be given a copy of this consent form, which includes the Authorization to Use and Disclose Protected Health Information for Research Purposes.

BY SIGNING BELOW, I WILLINGLY AGREE TO PARTICIPATE IN THE RESEARCH STUDY AND AUTHORIZE THE USE AND DISCLOSURE OF MY PHI AS DESCRIBED IN THIS FORM.

____

Printed Name of Participant

____ __________________

Signature of Participant Date

I have explained the nature and purpose, the potential benefits and possible risks associated with participation in this research study to the participant. I have answered any questions that have been raised and have witnessed the above signatures.

____

Printed Name of Person Obtaining Consent

____ ____________________

Signature of Person Obtaining Consent Date

Approved Date: July 22, 2024
Kaiser Permanente - Interregional IRB (KPiIRB)
